# The Effects of Physical-Chemical Evolution of High-Sulfur Petroleum Coke on Hg^0^ Removal from Coal-Fired Flue Gas and Exploration of Its Micro-Scale Mechanism

**DOI:** 10.3390/ijerph19127082

**Published:** 2022-06-09

**Authors:** Jie Jiang, Yongfa Diao

**Affiliations:** College of Environmental Science and Engineering, Donghua University, Shanghai 201620, China; jie.jiang@mail.dhu.edu.cn

**Keywords:** high-sulfur petroleum coke, Hg^0^ removal, pore structure, exposure of inherent S, controllable pyrolysis, chemical-mechanical activation, ReaxFF and DFT theory

## Abstract

As the solid waste by-product from the delayed coking process, high-sulfur petroleum coke (HSPC), which is hardly used for green utilization, becomes a promising raw material for Hg^0^ removal from coal-fired flue gas. The effects of the physical–chemical evolution of HSPC on Hg^0^ removal are discussed. The improved micropores created by pyrolysis and KOH activation could lead to over 50% of Hg^0^ removal efficiency with the loss of inherent sulfur. Additional S-containing and Br-containing additives are usually introduced to enhance active surface functional groups for Hg^0^ oxidation, where the main product are HgS, HgBr, and HgBr_2_. The chemical–mechanical activation method can make additives well loaded on the surface for Hg^0^ removal. The DFT method is used to sufficiently explain the micro-scale reaction mechanism of Hg^0^ oxidation on the surface of revised-HSPC. ReaxFF is usually employed for the simulation of the pyrolysis of HSPC. However, the developed mesoporous structure would be a better choice for Hg^0^ removal in that the coupled influence of pore structure and functional groups plays a comprehensive role in both adsorption and oxidation of Hg^0^. Thus, the optimal porous structure should be further explored. On the other hand, both internal and surface sulfur in HSPC should be enhanced to be exposed to saving sulfur additives or obtaining higher Hg^0^ removal capacity. For it, controllable pyrolysis with different pyrolysis parameters and the chemical–mechanical activation method is recommended to both improve pore structure and increase functional groups for Hg^0^ removal. For simulation methods, ReaxFF and DFT theory are expected to explain the micro-scale mechanisms of controllable pyrolysis, the chemical–mechanical activation of HSPC, and further Hg^0^ removal. This review work aims to provide both experimental and simulational guidance to promote the development of industrial application of Hg^0^ adsorbent based on HSPC.

## 1. Introduction

As the solid waste by-product of the delayed coking process in the oil industry, petroleum cokes are utilized according to their sulfur-containing which are represented by low-sulfur petroleum coke (<3% S-containing) [1] and high-sulfur petroleum coke (>3% S-containing) [2]. Low-sulfur petroleum coke (LSPC) has maturely been used as anode raw material for electrolytic aluminum [3] and graphite electrodes [4] in steel plants, which accounts for 56.7% and 3.94%, respectively. By contrast, the proportion of fuel in cement plants and power plants using high-sulfur petroleum coke (HSPC) was just 6.19% [5]. Although the share of HSPC is not dominated, its absolute output is still high which was 1100 million tons in 2015, and maintains a fast-growing pace [6], especially in those countries which imported raw oil from the Middle East. Investigated from crude oil-producing areas, such as Saudi Arabia and Iran, the sulfur in raw oil is usually higher than 2.5% due to conventional plant remains during crude oil generation in these specific geographic locations. The pollutant represented by SO_x_, which is released during the combustion of HSPC as fuel, has raised high concern for the atmospheric environment by global governments and organizations [7].

On the other hand, mercury also has caused global concern due to its high toxicity to the human body [8,9]. Mercury emissions are mainly derived from coal-fired plants which account for approximately 50% [10], especially in developing countries, such as China, which exist a high need for coal by 2018 [11]. The forms of the presence of mercury in the flue gas from coal-fired plants are the three, particulate-bound mercury (Hg^p^), oxidized mercury (Hg^2+^), and element mercury (Hg^0^) [12]. The first two forms of mercury can be almost completely captured through fabric filters (FF) and wet flue gas desulfurization (WFGD) devices respectively due to those characteristics [13]. However, conventional desulfurization and dust removal equipment cannot succeed to achieve acceptable performance in the removal of element mercury [14]. Although elemental mercury emission is a trace from the coal-fired industry, the accumulation and difficult removal properties, which are water-insoluble and volatile, have brought a worldwide threat to the biological environment and human health [15]. Therefore, compared to the high preparation cost of commercial activated carbon, which is usually modified by bromine [16], HSPC is a more attractive adsorbent for element mercury removal from coal-fired power plants due to its high sulfur-containing and also inexpensive cost. Furthermore, removing pollutants, which as the element mercury, with waste, which as HSPC, is the full use of artificial waste resources and meets the long-term requirements of carbon peaking and neutrality.

Compared to various raw materials for carbon-based activated adsorbents, such as fly ash [17,18], agricultural waste [19,20], and marine resources (sargassum and enteromorpha) [21], HSPC shows advantages as a promising candidate for its global production and relative stability of quality as low-economic-value waste. Furthermore, it is the sulfur in HSPC, which is the reason for the unsatisfactory selection as fuel, that is expected to oxidize Hg^0^ to HgS which is recognized as the most environmentally friendly form of mercury oxide. Yang et al. [22] prepared a regenerable Co-MF catalyst based on fly ash for Hg^0^ removal, where the catalyst played the oxidation role rather than the fly ash itself. Liu et al. [23] concluded that unburned carbon (UBC), Fe_2_O_3_, SiO_2_, AlO_3_, and CaO are the main reactive components in fly ash for Hg^0^ oxidation. In terms of production, HgO, which is the main oxidation production, is formed by metal oxides as well as mental positive ions. However, HgO is not so stable at high heating temperatures. Thus, the bromination [17,24] impregnation method for fly ash activation was conducted and high removal efficiency (almost 100%) was achieved. However, the same problem occurs. The final production, which is HgBr_2_, on the fly ash is readily volatile to air and leachable in water [25], which means secondary pollution during the coming disposal. For the raw materials of biomass, biomass-based activated carbon is usually prepared by improving pore structure, which is lacking for fly ash, and chemical activation. Thus, no matter which raw materials are used, chemical activation is the crucial activation process for element mercury removal, which plays a role in its oxidation and significantly improves its removal efficiency. The methods, including microwave activation, freeze-drying [26] and CO_2_ activation [27], and KOH activation [28], are used to optimize the pore structure. Spessato et al. [28] successfully obtained the ideal BET surface of Jatoba’s barks using KOH which were 2794 m^2^/g and 889 m^2^/g for SAC and RSAC, respectively. Apart from this, KOH activation during biomass pyrolysis [29] provided extra O-containing functional groups which benefit oxidizing element mercury. As such, the method combined KOH activation and pyrolysis seems an advanced activation technology for those materials which are expected to be porous for element mercury removal. Wu et al. [30] obtained a specific surface area of at most 3000 m^2^/g, which came up with commercial activated carbon. The fresh high-sulfur petroleum coke, which contained 6.2% total sulfur, was activated using KOH by Zhu et al. [31]. Although the specific surface area reached 1713.8 m^2^/g, the sulfur for element mercury oxidation almost lost and was not fully utilized. Therefore, this combined method is not a proper activation measure for HSPC due to its resource-wasting actions. Nonetheless, the actual ideal pore structure of petroleum coke has been a rare quantitative study for element mercury removal, including the parameters of particle size, specific surface area, and pore diameter as well as its shape. Due to the lack of sulfur which is in the favor of mercury, extra additives, such as SO_2_ [32] and NH_4_Br [33], for activation are needed to improve Hg^0^ removal performance. In comparison, HSPC has an outlook of few additives for activation due to sulfur-containing itself. Furthermore, mechanochemical modification is also used in the preparation of adsorbents for raw petroleum coke [34] because it is a simple operation process and ecologically safe [35]. This activation method should conduct more experimental exploration and mechanism research to adapt the directional preparation of HSPC. Meanwhile, the basic understanding of mechanisms of related modified activation and mercury removal using modified HSPC needs further studies which are the foundation for both pilot experiments and actual application.

In this work, most of the articles on activated high-sulfur petroleum coke for element mercury removal are critically reviewed. Herein, these are the main contents. Firstly, the developed porous structure of revised HSCP formed by the activation process, and also its contribution to Hg^0^ capture, are discussed. Secondly, the content and distribution of inherent sulfur in HSPC from various origins are identified. Afterwards, the exposure, transition, and re-fix of the inherent and additional sulfur during the activation process and their effects on Hg^0^ oxidation are discussed. In addition, O-containing and Br-containing functional groups of HSPC for Hg^0^ oxidation are investigated. Lastly, density functional theory (DFT) and reactive force field (ReaxFF) studies to explore the micro-scale reaction mechanisms of the activation process, including pyrolysis and mechanochemistry, of HSPC and its mercury removal are talked. Based on the effects of the evolution of pore structure and functional groups on Hg^0^ removal, this study select an activation method, which includes both controllable pyrolysis and chemical–mechanical activation, to obtain ideal pore structure and fully utilize the inherent sulfur, and even increase the content of O-containing and Br-containing functional groups in HSPC for Hg^0^ removal. Moreover, DFT and ReaxFF studies are expected to explore the micro-scale mechanism of both the proposed activation of HSPC and the pathways of Hg^0^ removal. This review is expected to provide guidance to achieving high efficiency, low cost, and high stability of activated high-sulfur petroleum coke for element mercury removal from coal-fired flue gas and its further actual industry application.

## 2. The Effect of the Evolution of Pore Structure on Hg^0^ Removal

The pore structure of raw HSPC is identified as poor for Hg^0^ adsorption due to its dense structure. In particular, the Brunauer–Emmett–Teller (BET) surface area of raw HSPC is tested to be less than 1.1 m^2^/g [36] and the total pore volume is almost zero [37]. It is the reason for the poor performance of pore structure on Hg^0^ adsorption and also the lack of positions for active sites. The current modification process to active petroleum coke, including only or combined chemical, pyrolysis, mechanochemistry, and KOH activation, almost improved the pore structure of HSPC to a certain extent.

**Figure 1:**
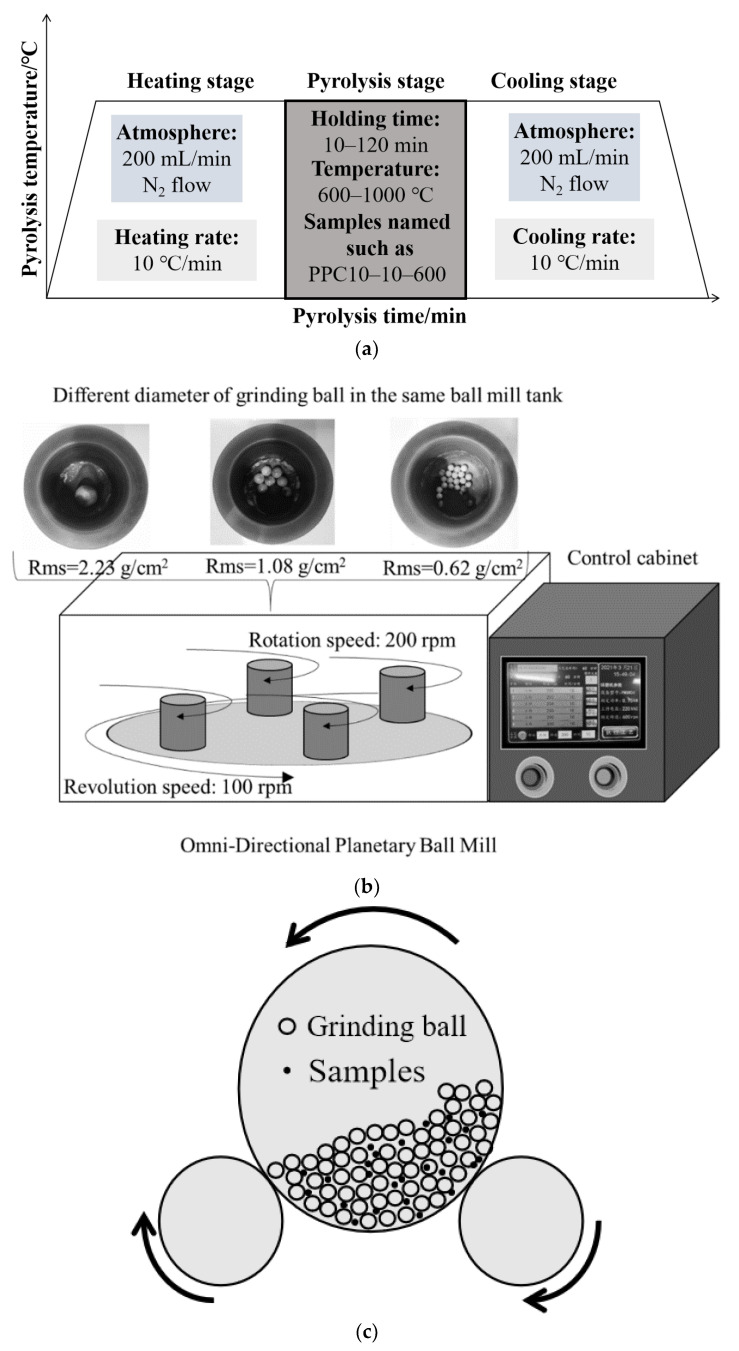
(**a**) Pyrolysis [34] Reprinted/adapted with permission from Ref. [34]. 2022, Chemical Engineering Journal; (**b**) 1st [34] Reprinted/adapted with permission from Ref. [34]. 2022, Chemical Engineering Journal; (**c**) 2nd mechanochemical activation technology.

For only chemical activation, Xiao et al. [38] used a chemical–mechanical bromination process [39] for brominating the petroleum coke sample, as shown in Figure 1c. Although the mechanical impregnation method presented well bromine loading, both specific surface area and average pore size were slightly reduced to 1.66 m^2^/g and 0.012 cm^3^/g, which was caused by the blockage of pores during the bromination process. It is believed that external mechanical force caused by grinding could not promote the development of rich pores. While this bromination method made an excellent performance of Hg^0^ removal, it was almost owing to the chemically loaded bromine of C-Br rather than the contribution of pore structure.

For only pyrolysis modification without other activation processes, there was little literature on only pyrolyzed petroleum coke for Hg^0^ removal. Although most research on pyrolyzed HSPC concentrated on gasification reactivity, the improvement and collapse of pore structure at different pyrolysis temperatures would provide valuable information for future studies. Li et al. [40] pyrolyzed high-sulfur petroleum coke, which was sieved to 83–165 μm, at the final temperature of 1223, 1473, and 1673 K with a pyrolysis heating rate of 10 K/min and holding time of 30 min under the atmosphere of N_2_. The test results showed that the BET surface area of the pyrolyzed samples was 15.62, 28.52, and 3.03 m^2^/g. The same trend occurred in the total pore volume that the smallest one was 0.0085 cm^3^/g and lower than half of that at 1473 K. The reason for it was the ash was melted at this high temperature and the melt blocked the micropore. Hence, excessive pyrolysis temperature was not conducive to the formation of an extremely rich pore structure. Lee et al. [36] divided the pyrolyzed PC, which was heated up to 1100 °C under N_2_ within 30 min, by the particle size of 200, 400, and 500 mesh. The results showed that the smaller particle size led to the larger specific surface area where the largest one was 11.7 m^2^/g. Compared to other activated carbon (AC) with a specific surface area of more than 1000 m^2^/g, such as HGR, SH-S, and Norit GL, PC-400 had a promising Hg^0^ removal performance while its specific surface area was only 1/100 of that of these three commercial AC. This work concluded that the reason was that mesopores above 50 Å were developed for the PC400 after pyrolysis and sulfur separated from carbon and adhered to macropores in the process of PC pyrolysis. On the contrary, the quantity of sulfur adhered to the micro-pores was smaller than that in the macro-pores. It should be noted that the formation of pores in commercial AC is almost that of micropores. Hence, although the chemical oxidation offers a more active and stable fix for Hg^0^, the advanced pore structure with different proportions of macropores, mesopores, and micropores should be explored.

For the combined pyrolysis and mechanochemistry activation method, Ma et al. [34] crushed the samples with particle sizes lower than 100 mesh and carried on the only pyrolysis (Figure 1a), mechanochemical activation (Figure 1b), and the combined pyrolysis and mechanochemical activation experiments. The final temperature and holding time of pyrolysis parameters were concerned. The omni-directional planetary ball mill was selected for mechanochemical activation, which was the same method as Xiao et al. [38] with different equipment. It was observed that the largest specific area of 31.77 m^2^/g, the total pore volume of 0.027 cm^3^/g, and the smallest mean pore diameter of 3.39 nm occurred in PPC10-60-800. The influence of final pyrolysis temperatures was the collapse of pore structure happened at 900 °C, which was approximately lower than 200 °C compared to other experiments. In terms of only mechanochemical activation, the largest specific surface area reached 5.93 m^2^/g and the mean pore diameter came to 8.1 nm. However, its development was not considerable as that of pyrolysis. Combined with the results of Xiao et al. [38], it can be concluded that the significant contribution of mechanochemical activation is mainly for the chemical loading rather than the improvement of pore structure. The mercury removal efficiency of only pyrolysis, only mechanochemical activation, and the combined pyrolysis and mechanochemical activation were 29.15%, 25.68%, and 27.8%, respectively, which indicated that the improved pore structure could act as conventional functional groups, such as C–O, C–O–C, and C=O, for mercury removal. Hence, the optimal pore structure can improve the mercury removal performance of HSPC to a certain extent which cannot be vague and random identification. It is noted that pyrolysis parameters are raised concern for the development of optimal pore structure for Hg^0^ adsorption and better chemical loading for Hg^0^ oxidation, such as atmosphere, final temperature, heating, and cooling rate, holding time, and even the mesh selection for controllable pyrolysis. Zhao et al. [41] reported the largest specific surface area of pyrolyzed biomass coke by pyrolysis parameters of 7% O_2_, 800 °C, and the heating rate of 10 K/min. Moreover, the micropore structure was obtained with an average pore diameter of 1.93 nm. Therefore, sufficient attempts and explorations of pyrolysis parameters should be conducted in the future.

**Figure 2:**
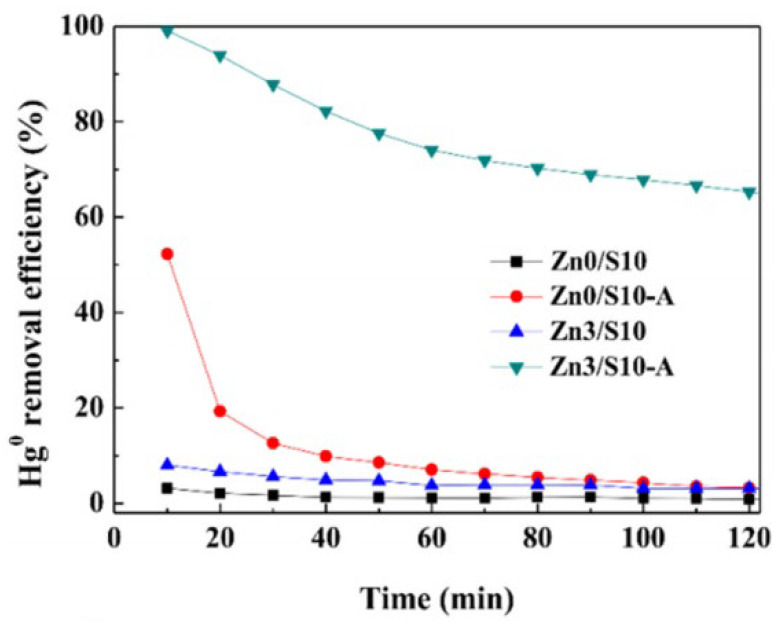
Hg^0^ removal performance of different ZnS sorbents at different temperatures [37]. Reprinted/adapted with permission from Ref. [37]. 2019, Fuel Processing Technology.

For the combined pyrolysis and chemical activation method, Huo et al. [37] prepared AC from HSPC with zinc nitrate in the following process, 2 h ultrasound mixing, 12 h dry at 60 °C, 5 h calcine, and physical activation at high temperature. The specific surface area of the ZnS/AC sample was 0.21 m^2^/g, which also indicated that chemical activation could not well improve the pore structure [38]. The activation parameters that 950 °C, 40 vol% water stream, and 2.5 h activation time were selected. Then, the optimal specific surface area, total pore volume, and average pore diameter were obtained, which were 235.84 m^2^/g, 0.139 cm^3^/g, and 3.9 nm, respectively. The comparisons of four samples on Hg^0^ removal before and after these two activation process were given (Figure 2). Apparently, Zn3/S10-A sample had the excellent Hg^0^ performance at begin, and then decrease to 65% within 2 h, which is due to the developed pore structure and ZnS as active sites to oxidation Hg^0^. The Zn0/S10-A sample also showed a much higher Hg^0^ removal efficiency compared to the other two samples without improved pore structure. This work further explained the reason for the decline of the Hg^0^ removal efficiency of Zn0/S10-A, which is the consumption of a small part of released sulfur during pyrolysis. However, the specific contribution between pore structure and the active sites were not be mentioned. In other words, it is vague to distinguish these two contributions for Hg^0^ removal and also identify their interaction. In addition, although the ZnS could not improve the pore structure, Hong et al. [42] pointed out the specific surface area of AC was developed up to 1475 m^2^/g by ZnCl_2_. The huge difference in the hole-enlarging effect of zinc-based additives may be the final pyrolysis temperature, which was due to the decomposition and vigorous movement of zinc-based additives at higher temperature (>500 °C). Meanwhile, the collapse of the pore structure was also 900 °C which was the same as Ma et al. [34]. It is noted that the ZnCl_2_ activator has the potential to produce mesoporous AC [43], while some other activators such as KOH, NaOH, and Na_2_CO_3_ are beneficial to the production of microporous AC [44]. Thus, the mesoporous rate of samples activated by ZnCl_2_ is high, reaching approximately 75%. It is assumed that a rich mesoporous structure might better promote further chemical loading than a microporous structure. In addition, the preparation mechanism by ZnCl_2_ was proposed [42] (Equations (1) and (2)).


(1) $${\mathrm{ZnCl}}_{2}+H_{2}O\to H[{\mathrm{ZnCl}}_{2}(\mathrm{OH})]$$

(2) $$H[{\mathrm{ZnCl}}_{2}(\mathrm{OH})]\to \mathrm{ZnO}+2\mathrm{HCl}↑$$

For the combined mechanochemistry and chemical activation method, Ma et al. [45] selected S/FeS in the mechanochemical modification that parameters were based on their previous study [34]. It was found that FeS600-60-25, which contained more FeS than other samples, had the largest specific surface area and the smallest mean pore diameter of 27.33 m^2^/g and 3.72 nm, respectively. This was higher than that of S600-60-17 that which the specific surface area and mean pore volume were 2.69 m^2^/g and 13.141 cm^3^/g, respectively. However, the mercury removal efficiency of S600-60-17, which was 98.6%, was much higher than that of FeS600–60–25, which was just 63%. It should be noted that, this study, as well as the study of Xiao et al. [46], did not support the consensus that larger specific surface area, larger total volume, and smaller mean pore diameter led to higher mercury removal efficiency. In general, mechanochemistry and chemical activation methods could provide more exposed carbon to load C–S or C–Br. Considering the chemical surface loading of modifiers, the optimal pore structure could promote more chemical loading to increase the mercury removal capacity of modified HSPC.

For combined pyrolysis and KOH activation, Zhu et al. [31] proposed a two-step preparation method of columnar sulfur-impregnation in which KOH activation under 750 °C for 1 h played an effective step to promote the development of pores. When the weight ratio of KOH solution to raw petroleum coke came to 3:1, the specific area and the total pore volume dramatically increased to 1713.8 m^2^/g and 0.8615 cm^3^/g, respectively. This is due to the etching effect by free potassium decomposed from KOH [47]. However, excessive KOH solution led to a decrease in micropore volume proportion, which was 84.78% when its ratio was 3:1 and 97.12% when its ratio was 2:1. At the same time, the mean pore diameter reached over 2 nm in the ratio of 3:1 compared to that of 1.8 nm in other conditions. Furthermore, the following sulfur-impregnation process could broaden the micropores to mesopores because the sulfur was mainly deposited on the carbon matrix in micropores and might cause blockage. Although the expanded specific surface area and largest micropore volume were obtained, the experiments which illustrated the only contribution of its physical structure to adsorption Hg^0^ were not conducted. Hence, the contribution of pore structure should be paid more attention to Hg^0^ capture, regardless of physical adsorption or offering positions for chemicals loading to oxidize Hg^0^.

Learned from the above analysis, although mercury oxidation mainly relies on chemical positions, the physical adsorption cannot be ignored. The physical adsorption process includes the capture of Hg^0^ to the surface and even into the inner channels, which then form Hg^p^ which could not increase the valence of mercury. When the porous material to remove Hg^0^ was discussed, the transportation of Hg^0^ into the inner channels remained to be discussed as the same condition as surface physical adsorption. In other words, the movement of Hg^0^ into HSPC is ignored. Note that the particle size of PC-based AC was manually ground within 0.1 mm to 1.65 mm, which should be considered to affect the physical adsorption. Selected in accordance with the relationship of Hg^0^ and pore diameter, the macropore is recognized as the initial entrance window and the mesopore is the channel of Hg^0^ transportation, while Hg^0^ could be stopped over the micropore, respectively. However, there are few studies that concentrate on the relationship between multi-level pores and the performance of physical adsorption for Hg^0^. Meanwhile, it is evident that the big share of the developed mesopores is beneficial for internally decomposed sulfur to load on the surface and improve the Hg^0^ removal capacity. Thus, it is comprehensive to determine the optimal pore structure due to this coupling effect on both physical adsorption of Hg^0^ and chemical loading of additives. In other words, the optimal share of macropore, mesopore, and micropore rather than filled micropores, as well as the arrangement of these pores, should be studied. Among these activation methods, pyrolysis with selected parameters, called controlled pyrolysis, is a candidate to modify the pore structure of HSPC, while KOH activation loses almost sulfur content. Although the binding strength of physical adsorption is relatively weak compared to chemical adsorption, the optimal pore structure will be beneficial to the higher Hg^0^ adsorption capacity, as well as the following chemical activation. As such, controllable pyrolysis, including various parameters mentioned above, with additives to expand pore tunnels, such as Zn and Fe, will be hoping to develop the optimal pore structure. In addition, the relationship between parameters of pore structure, adsorption capacity, and further chemical loading should be explored to evaluate the optimal pore structure.

## 3. The Effect of the Evolution of Functional Groups on Hg^0^ Removal

Apart from the contribution of pore structure, the role of the chemical active sites, which are always chemically loaded on the surface of HSPC, is essential to oxidizing Hg^0^. Reviewing current studies on modified HSPC for Hg^0^ removal in flue gas, modification of additives were element sulfur, sulfur compounds, and liquid bromine. The corresponding productions mainly included HgS (red and black), HgBr, HgBr_2_, and also HgO with the oxidation by sulfur-containing, bromine-containing, oxygen-containing functional groups. Herein, the evolution of these surface functional groups with modifiers, and also their effects on performance and mechanism as active sites for Hg^0^ removal are discussed.

### 3.1. Sulfur-Containing Functional Groups for Hg^0^ Removal in HSPC

Compared to other raw carbon-based materials for Hg^0^ removal, the natural advantage of HSPC is the rich composition of sulfur exists. In addition, the sulfur-additives also offered extra sulfur active sites on the surface of modified HSPC for Hg^0^ oxidation.

#### 3.1.1. The Identification of Inherent Sulfur in HSPC

It is the surface and internal sulfur in HSPC, which contains elemental, inorganic, and organic sulfur, that is expected to be fully utilized for Hg^0^ oxidation. Although the relatively high and stable production of raw HSPC is all over the world, it is observed that the quantity, formation, and distribution of sulfide in HSPC samples are not so stable, which is not beneficial for the actual industry application. Therefore, specific and comprehensive identification of the formation and distribution of sulfur in HSPC is necessary for controllable activation and even the new method of mechanochemical modification for mercury oxidation. This section firstly reviewed the sulfur content of HSPC used from most published papers on Hg^0^ removal and some studies on gasification, both of which contain pyrolysis, partial combustion, and chemical activation.

In general, as data was collected, the total sulfur content of selected HSPC from different origins presented significant diversities. It is shown in Table 1 that the sample HSPC14 from Xiamen, China, had the highest total sulfur content which was higher than 8%, while the total sulfur content of HSPC4 from Jiangxi, China, was slightly higher than 4%. Moreover, there are certain fluctuations in the sulfur content of HSPC samples among different countries. Hence, various HSPC with different sulfur content should be activated by a controlled method to ensure mercury removal performance. In addition, the XRF method or ultimate analysis is aimed at detecting the total sulfur content in HSPC, while the XPS method is recognized for distinguishing the forms of surface sulfur. Although the surface functional groups are seen as the active sites for Hg^0^ oxidation, the internal sulfide in the bulk, regardless of organic and inorganic sulfur, shows its potential, which cannot be ignored. 

**Figure 3:**
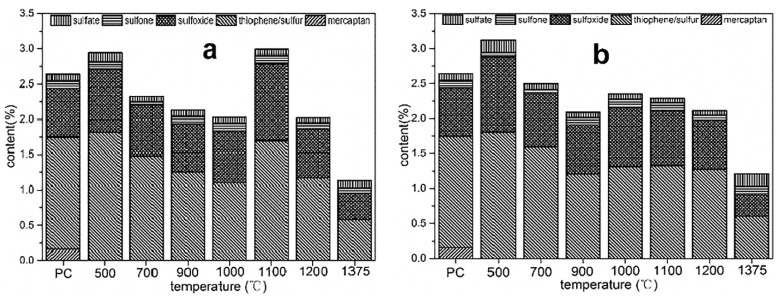
The distribution of sulfur forms on the surface of petroleum coke varies with temperature under different heating rates; (**a**) 10 K/min; (**b**) 30 K/min [49]. Reprinted/adapted with permission from Ref. [49]. 2021, Fuel.

Furthermore, the controlled preparation method, including controllable pyrolysis with different pyrolysis parameters and chemical activation, should be based on the identification of various forms of HSPC rather than only total sulfur content. Xiao et al. [38] found that over 90% of sulfur was thiophene and organic sulfide (R-Sn-R), and the rest 10% was in the form of oxides, such as sulfate. She et al. [48] analyzed the XPS spectra which suggested that more than 94.40% of the inherent sulfur existed as thiophene. Meanwhile, sulfoxide, sulfone, and sulfate were detected in very small quantities. Yu et al. [49] detected the content of total sulfur and surface sulfur by element analyzer and XPS method, respectively. It was found that the surface content of S in the original HSPC sample was about 2.64% which was far lower than the results of element analysis which was 4.2%. This evidently showed a relatively big difference in the content of sulfur between the surface and the interior of HSPC. The specific forms and corresponding containing shares of HSPC pyrolyzed in the heating rate of 10 K/min and 30 K/min were given out shown in Figure 3 [49]. In detail, the surface S of the raw HSPC includes various forms including thiophene, sulfoxide, mercaptan, sulfate, and sulfone. Among them, thiophene and sulfoxide are the two primary forms with proportions of about 60% and 26.5%, respectively. However, Ma et al. [34] gave out the different detection results that the main form of S was sulfide (64.44%) and the content of thiophene was just 32.91%. 

As such, it can be concluded that, regardless of total sulfur content, surface sulfur content, forms, and shares of sulfur, it is so different for selected HSPC samples from origins. These complex content and forms in various HSPC samples from origins will lead to the mercury removal performance by activation method uncontrollable when the sample changes. Therefore, a controllable modification and activation method should be determined. Furthermore, noted that thiophene dominated the content of surface sulfur in most samples, which can be exposed to be active sites for Hg^0^ oxidation for the saving of additives or to improve the mercury removal capacity with the same amount of additives used.

#### 3.1.2. The Evolution and Contribution of Sulfur in HSPC

Present modification methods for HSPC activation mainly include pyrolysis and chemical activation. Under these activation methods, the transformation and utilization of sulfur species in HSPC were observed. Commonly, the pyrolysis could have effects on the inherent sulfur, while extra S-containing additives offer additional S-containing functional groups on the surface of HSPC for Hg^0^ removal.

Xiao et al. [55] proposed a new activation process that the inherent thiophene could enhance bromine binding and then increase the mercury removal of HSPC while the stable thiophene almost cannot oxidize Hg^0^. In specifics, the experimental observations demonstrated that HgBr and HgBr_2_ were approaching binding on the carbon site next to the S atom. The synergetic effect of the inherent thiophene sulfur and loaded bromine enhanced the Hg^0^ removal efficiency of this adsorbent. Despite this, it is critical to better expose the stable thiophene in HSPC to provide more active sites for Hg^0^ removal. Yu et al. [49] explored the evolution of the speciation of sulfur in both gas and solid phase during pyrolysis where the pyrolysis temperatures are in the range of 500–1375 °C. The desulfurization ratio increased with increasing temperature or decreasing heating rate. The results of the transformation of individual S species showed in Figure 3. It is observed that both thiophene and sulfoxide increased under 500 °C which was due to the bulk S combined with surface free radicals or small molecular hydrocarbon bonds to form thiophene and sulfoxide structures. Unfortunately, thiophene and sulfoxide decomposition and transformation to S-containing gas, such as H_2_S, SO_2_, CH_3_CH, and COS, are under 500–900 °C. These losses of sulfur from solid to gas were not conducive to further mercury removal because of the decrease of S-containing functional groups on the surface. However, at stage III (900–1100 °C), there appeared a second increase in thiophene and sulfoxide. There were two possible reasons proposed for this phenomenon. One is that the competitive reactions of C- or N-containing free radicals for the combination of H- or O-containing free radicals existed. The other is that the rate of S migration towards the particle surface might be faster than that of S release into the gas environment. The dramatic decomposition of thiophene and sulfoxide happened at high pyrolysis temperatures higher than 1100 °C, which was consistent with previous studies. Although this research could not carry on a mercury removal test, the transformation of the S-containing groups and the released S-containing gas components give the help for further modification of HSPC. It is noted that the released S-containing gas during pyrolysis, such as SO_2_, was selected to activate HSPC [48].

**Figure 4:**
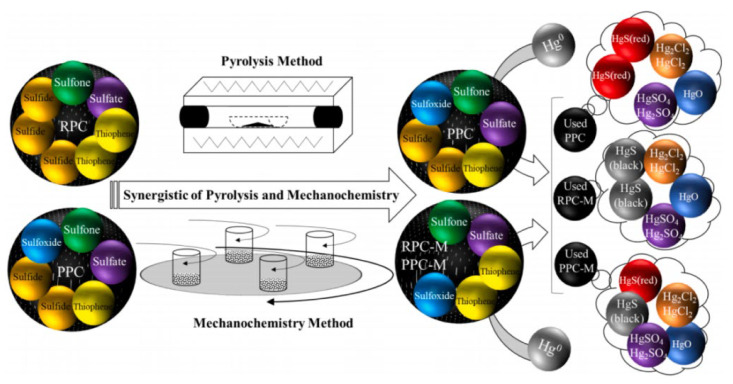
Activation and mercury removal mechanisms of RPC, PPC, RPC-M, and PPC-M [34]. Reprinted/adapted with permission from Ref. [34]. 2022, Chemical Engineering Journal.

Ma et al. [34] explored the effect of pyrolysis and mechanochemical activation method on the transformation and utilization of surface S-containing groups of HSPC. The findings illustrated that the pyrolysis would slightly accelerate the transformation of the S-containing groups. However, specifically on the transformation of thiophene, the share of thiophene decreased continuously from 32.91 at. % to 23.78 at. % during the increasing final pyrolysis temperature (600–1000 °C). Both the content and trend were significantly different compared to the study of Yu et al. [49]. According to the explanation of Yu et al. [49] for the twice increase of thiophene, the selected particle sizes were concerned. The particle size selected by Ma et al. [34] was lower than 150 μm, while that of Yu et al. [49] was below 100 μm. It seems that smaller particle size would contribute to the release of bulk S to the surface of HSPC which could improve the utilization of the total S-containing group. Hence, the uncontrollable selection of HSPC would lead to different activation results, which is not conducive to further mercury removal. In other words, the effect of the pyrolysis process on the transformation of sulfur in HSPC is still vague. In terms of the effects of the mechanochemical activation method, it was interesting that this process transformed all sulfide into thiophene and sulfoxide. Based on the results of the Hg-Temperature Programmed Desorption (Hg-TPD) test, it can be inferred that the sulfur form produced by the pyrolysis method might be more likely to form HgS (red), while which produced by mechanochemical method would be more likely to form HgS (black), shown in Figure 4. Considering the opposite mercury removal performance of PRC-M-15M and RPC-M-15S, sulfone and sulfoxide played the important role compared to other S forms. It should be noted that, pyrolysis and mechanochemical activation of HSPC only increased the mercury removal performance by 13% compared to raw petroleum coke. Thus, extra additives are needed to further activate it with more active sites.

While the method of KOH activation to improve rich micropore structure was conducted for biomass [29] and petroleum coke [31], the sulfur in HSPC would be cleared up, which could not be the full utilization of sulfur in HSPC for mercury removal. Shan et al. [53] further gave out the transformation of sulfur species during KOH activation of HSPC samples collected from Zibo in China and America to ensure the results are reliable. The reasons for the desulfurization were the three. At low temperature (<623 K), part of the sulfur was pyrolyzed and reacted with melting KOH. At high temperature (>623 K), KOH converted into K_2_O and H_2_O, and then both H_2_O and K_2_O reacted with the residual sulfur, and also reacted with carbon to form potassium vapor and K_2_CO_3_, which keep reacting with sulfur compounds. Lastly, the excessive alkali could absorb sulfur and the temporary equilibrium would be destroyed, thus the sulfur in HSPC keeps released. Moreover, KOH activation increased the reactivity of carbon in the external porous layer and the K_2_CO_3_ formed in the initial pores produced by the activation of C/KOH reactions could react with the carbon inside the pores, which would further develop the porosity, so the sulfur molecules inside the HSPC could be exposed to melting KOH and then the reaction becomes to access. In terms of the production, there was no COS and CS_2_ in the gas phase during the activation process, which differed from the pyrolysis of HSPC, because the reaction was prevented by KOH. Further thermodynamic analysis based on the Benson group contribution method illustrated that thiophene tended to generate an equal amount of sulfate and sulfide but more SO_2_ than H_2_S, and sulfoxide tended to generate more SO_2_ than H_2_S and more sulfate than sulfide. Except for KOH activation, the inherent sulfur of petroleum coke also could be accessed by nitric acid functionalization [54]. The proposed reaction pathway for the oxidation of dibenzothiophene [56] was seen that sulfur in the thiophene groups could be oxidized to sulfone which could be further oxidized to sulfonic and hydroxyl groups in the presence of water.

In general, the understanding of exposure and transformation of inherent S-containing groups, especially thiophene and sulfoxide, for Hg^0^ removal is insufficient. While pyrolysis is the common method to revise HSPC, the pyrolysis parameters mentioned before should be carefully considered due to the unpredictable fluctuation of sulfur content, forms, and distribution of the collected HSPC samples. In addition, some studies tried to activate HSPC with S-containing additives for mercury removal.

Zhu et al. [31] almost cleaned up the conventional sulfur in petroleum coke by KOH activation and further conducted element sulfur impregnation with a S/samples weight ratio of 1:2 under 400, 500, and 600 °C. It was found that the higher impregnation temperature would lead to better Hg^0^ removal efficiency of 60%, which could be explained that shorter chains of elemental sulfur were generated with increasing impregnation temperature. In addition, the reactive sulfur chains seemed to prefer to react with the carbon matrix rather than the load on the carbonaceous surface. When the inlet mercury concentration increased to 522.3 μg/m^3^, the mercury removal performance could maintain almost the same as the condition of 226.6 μg/m^3^, which was 60%. This could be due to the increased driving force with higher inlet mercury concentration that migrates more mercury into deeper active sites. This founding places higher demand on the pore structure of HSPC, which justifies the importance of the pore structure mentioned above. The result of the Hg-TPD test showed that the production of HgS was generated by the bulk sulfur (10%) and nonoxidized sulfur (8%). 

**Figure 5:**
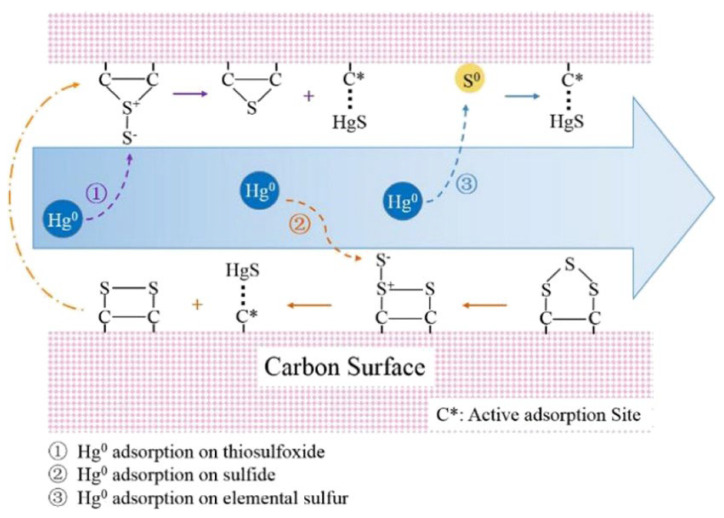
Possible reaction scheme of mercury capture by nonoxidized sulfur species in SO_2_ activated cokes [48]. Reprinted/adapted with permission from Ref. [48]. 2020, Energy and Fuels.

She et al. [48] emphasized the contribution of nonoxidized sulfur species, which contains reduced sulfur, sulfide sulfur, and elemental sulfur, in three kinds of SO_2_-activated HSPC for Hg^0^ removal. The SO_2_ activation processes were chosen in the pyrolysis step at 650 °C and the cooling stage, where the concentration of SO_2_ was maintained at 30%. The transformation of sulfur in SO_2_ was in accordance with the SO_2_ reduction mechanism proposed in terms of porous materials that SO_2_ molecules were firstly incorporated into the carbon matrix in the form of oxidized sulfur-containing intermediates by interactions with the active sites, and then the oxidized sulfur-containing intermediates decomposed to nonoxidized episulfide intermediates and CO_2_. Episulfides initiated the transportation of sulfur out of the carbon matrix with an equilibrium sulfide–disulfide–trisulfide–S_2_ system. It was observed from the XPS analysis that the elemental sulfur only occurred when SO_2_ activation was provided in the cooling stage. Although the share of elemental sulfur was only 0.74 at. %, which was much less than that of sulfide and thiophene, this added activation process gave out an effective method to provide more active sites for Hg^0^ oxidation. Under these circumstances, the revised HSPC, called PC–SC, showed a mercury capacity of 622 μg/g at 80 °C. Unfortunately, elemental sulfur suffered from severe temperature sensitivity for mercury binding. Furthermore, as shown in Figure 5, apart from being oxidized by elemental sulfur, mercury preferred to combine with thermal-stable organic sulfide in HgS and Hg-SR. Considering the mercury adsorption reactivity rank that PC–NS > PC–S > PC–SC, the TPD result suggested that various sulfur species played different thermal stability when that affected Hg^0^ adsorption capacity. In addition, She et al. [32] found that compared with nonoxidized and oxidized sulfur forms, the reduced sulfur forms showed a rather significant correlation with the mercury adsorption performance of SO_2_-impregnated samples. Furthermore, Reddy et al. [57] reported that sulfonated carbons are a potential candidate for Hg^0^ removal due to their –SO_3_H groups. Thus, the behavior of various sulfur forms in different activated HSPC-based adsorbents is so complex that it needs to be further determined.

The additives of element S and FeS, as modifiers, for activation of HSPC showed a certain disparity in mercury removal efficiency, which was with the same theoretical sulfur content of 17%. The mercury removal efficiency of S-revised HSPC was more than twice that of the FeS-revised sample which was 45% [45]. The functional group of C≡C and C≡N could be generated by mechanical force while C=C and C=N would be just influenced by the additives of S and FeS. However, C–S was not changed in the mechanochemical modification with S addition. It is a new founding that C≡C and C≡N play a role in the increasing mercury removal performance under the treatment of the single mechanochemical process. The surface sulfur content by the XPS test was much lower than the theoretical sulfur content, which indicated the existence of inner sulfur. These inactive inner sulfur, regardless of fresh or revised samples, shows a certain potential to improve mercury removal efficiency. In addition, the partial additive of FeS was oxidized as Fe_2_(SO_4_)_3_. As shown in Table 2, combined with the mercury removal performance, both activated HSPCs modified by S and FeS had a good agreement with the previous conclusion of affinity with mercury that compared with sulfide and sulfate, elemental sulfur, sulfoxide, and sulfone have more positive effects on mercury removal [32]. In addition, Reddy et al. [58] pointed out that CuS-doped carbon had a higher Hg^0^ removal performance of 23 mg/g compared to that of FeS-doped carbon, which was attributed to better dispersion of active phase (CuS), lower diffusional resistance for mercury, and also activity of active phase. Abraham et al. [59] firstly demonstrated the utilization of porous sulfur co-polymers as reactive adsorbents for capturing Hg^0^ due to their controllable pore structure and high content of sulfur. However, thiophene is not divided with the identification of elemental sulfur by the XPS test. Provided the binding performance of thiophene and element S, it is essential to quantitatively distinguish these two sulfur forms changed by advanced technology while the great share of thiophene on the surface of HSPC. The results of Hg-TPD illustrated that HgS (black) was the main oxidized mercury form and it could be assumed that some sulfur forms played the key role rather than Fe. It also emphasized the critical factor of sulfur in HSPC for mercury oxidation. Interestingly, this research proposed a new Hg^0^ removal pathway that the element sulfur could be released to gas over 200 °C, and then the S _(g)_ would directly react with Hg^0^ to generate HgS as Br [60]. These HgS, presented as fine solid particles, also could be captured by HSPC samples. Although this new reactive pathway needs strict reactive conditions and the amount of released S_(g)_ is not dominant, the mercury removal mechanism of S-containing activated HSPC, even other S-containing activated carbon-based adsorbents, is expected to be completed with more experimental and simulation evident.

Apart from sulfur-containing additives as modifiers, there was one study concentrated on the inherent sulfur in HSPC for Hg^0^ removal. Huo et al. [37] noticed that the local sulfur in HSPC could be utilized for element mercury removal by in-site method with the additive of zinc nitrate. The result of XRD showed the generation and transformation process of ZnO and ZnS, where the peaks of ZnO disappeared and ZnS reached the highest level under 750 °C. The S of ZnS was from the local S in the raw HSPC. Although the water steam could improve pore structure, this method would bring out some sulfur to form sulfur-containing gas. In addition, the volatilization of Zn also could lead to a certain loss of ZnS under high temperatures. It was proved that HgS is the product that contained both β-HgS (black) and α-HgS (red). However, few further mechanisms of transformation of conventional sulfur, such as organic and inorganic sulfur, were presented.

Overall, considering the binding affinity of Hg^0^ and sulfide and the environmentally friendly product of HgS, regardless of HgS (black) or HgS (red), the natural advantage of HSPC, which is high content of inherent sulfur, makes it promising for Hg^0^ removal. Most previous studies focused on the S-containing additives to modify this raw material to try to seek higher mercury removal efficiency rather than the full utilization of local sulfur. Provided the numerous sulfur forms existed on the surface and even in the internal adsorbent collected from origins, how to expose invalid sulfur, especially thiophene, to form active sites for Hg^0^ removal is a critical scientific problem. For it, the evolution of sulfur and its oxidation on Hg^0^ of different samples should be determined.

### 3.2. Oxygen-Containing Functional Groups for Hg^0^ Removal

Apart from the active sites of S, the oxygen-containing functional groups on the surface of mercury adsorbent also show a significant effect [61,62]. Actually, there is a little oxygen-containing functional group on the surface of raw HSPC, so extra activation for the generation of effective oxygen functional groups is necessary. 

Lee et al. [36] pyrolyzed the fresh PC samples in the pyrolysis and cooling atmosphere of N_2_. It concluded that oxygen took part in the generation of SO_2_ and CO_2_ rather than the oxidation of Hg^0^ because there was no HgO in the product detected. It can be assumed that the single pyrolysis process would not make the local oxygen become active functional groups for mercury oxidation. Xiao et al. [38] found the oxygen atomic concentration of about 7 at. % by XRF test after modification of the chemical–mechanical bromination process. The XPS analysis illustrated that HgO occurred and there existed positive oxygen-containing functional groups due to the chemical–mechanical bromination process. However, Ma et al. [34] could not find the product of HgO, which was consistent with the conclusion of Lee et al. [36]. The specific oxygen was binding with S to form S=O which did not perform oxidation of mercury. Another study, which concentrated on the combined pyrolysis and mechanochemical method, also proved that pyrolysis could lead to the disappearance of C–H, C–O, C–O–C, and C=O and mechanochemical activation has little influence on these functional groups [34]. However, the oxygen-containing functional group occurred by the XPS test, and the product of HgO, HgSO_4_, and Hg_2_SO_4_ was confirmed by Hg-TPD. It is noted that, though the modification method of both studies used was mechanochemical activation, different selections of mechanical equipment and even chemicals could lead to the unexpected and uncontrollable activation consequent, also the same conclusion applies to the activation method of pyrolysis.

**Figure 6:**
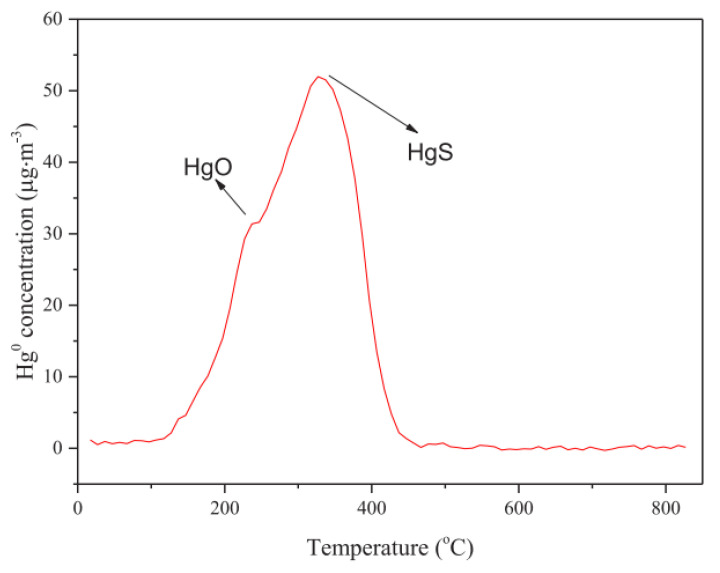
TPD results of CSAC after mercury adsorption [31]. Reprinted/adapted with permission from Ref. [31]. 2020, Energy and Fuels.

Zhu et al. [31] pointed out that the surface oxygen functional groups reached more than 24 at. % by KOH activation and SO_2_ impregnation, which was significantly more than that of Xiao et al. [38]. In detail, the oxygen functional groups were enriched by adding carbonyl (C=O) and carboxyl/ester (COOH/C(O)–O–C) functional groups. The quantitative classification of oxygen-containing functional groups is distinguished in Table 3, where beneficial oxygen functional groups were C=O and COOH/C(O)–O–C. Furthermore, as shown in Figure 6, the concentration of HgO, one of the products, was approximately 30 μg/m^3^ while that of HgS was slightly more than 50 μg/m^3^. Thus, oxygen-containing functional group for mercury removal is essential and cannot be ignored. 

As discussed, it is difficult to utilize the conventional oxygen-containing functional groups or make them active where the amount of that is few on the surface of HSPC. Pyrolysis is a complex method to improve both structure and the surface functional group for mercury removal. Combined with the aim of optimal pore structure, pyrolysis with adjusting parameters including temperature, heating rate, atmosphere, holding time, and so on, is a hopeful activation for adding the oxygen-containing surface functional groups. Zhao et al. [41] set the pyrolysis of 7% O_2_ and 15% CO_2_ under the pyrolysis temperature of 500−800 °C with the heating rate of 5, 10, and 15 K/min for biomass. The result of FTIR showed that the functional groups on the surface of the samples mainly exist in the form of O–H, C–O, and C–O–O–. The content of elements and carbon-containing functional groups on the surface of the typical BC samples was shown in Table 4. In particular, the content of C=O on the sample surface is more than that of the BC prepared under the other two pyrolysis atmospheres (N_2_, or N_2_ + 15% CO_2_). Thus, the selection of pyrolysis atmosphere of O_2_ could be the best choice. In addition, the introduction of O_2_ in the pyrolysis process could be called partial combustion. In comparison, the amount of C=O in this study, which was considered the most positive oxygen-containing functional group, was slightly lower than that of treatment of KOH [31]. Therefore, sufficient experiments on pyrolysis parameters to successfully active raw HSPC by improving the effective oxygen-containing surface functional group for Hg^0^ removal are essential and needed.

### 3.3. Bromine-Containing Functional Groups for Hg^0^ Removal

Hong et al. [63] firstly modified the pre-treated petroleum coke with a bromine-containing additive which was NH_4_Br. The result of EDS showed the uneven loading of Br on the different sites of the surface. The mercury removal capacity reached 24.9 μg/g with almost 100% removal efficiency, which was nearly 30% more than that of PAC. In addition, the introduction of 1% NH_4_Br performed the best compared to that of 3% and 5%. It was assumed that excessive Br could block the micropores, and even react with other chemicals to make them collapse. However, the products of this revised adsorbent were not presented. 

**Figure 7:**
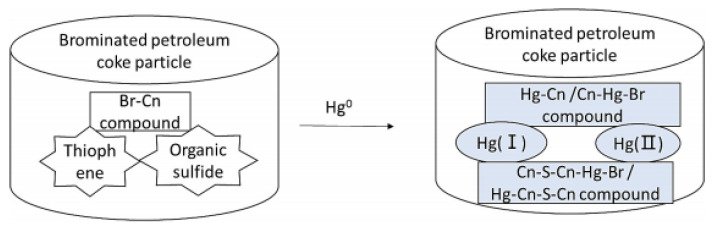
Diagram illustrating mechanism of Hg^0^ absorption on brominated petroleum coke surface [46]. Reprinted/adapted with permission from Ref. [46]. 2017, Carbon.

Xiao et al. [46] carried out a chemical–mechanical bromination process for HSPC that which stable bromine loading was found up to 200 °C. The mercury removal efficiency of revised PC at 1% and 3% bromine was almost 100% which was slightly higher than that of Br-Norit at 11% bromine. This Br-revised PC was more promising to replace Br-Norit due to its cost-saving of fewer Br-additive used. Furthermore, an interesting phenomenon of the synergetic effect of Br and S of Br-revised PC on mercury removal was found. In detail, the result of XPS showed that compared with Br-PC2, the binding energies in the range of 72–76 eV of Br-PC1 may be attributed to bromine binding on the carbon near S atoms. On the other hand, it could be concluded that a higher inherent sulfur content, especially thiophene, in Br-PC1 would enhance more active sites where bromine bonded. The mercury removal performance of revised HSPC at 1% bromine [46] was about 30% more than that of modified HSPC at 25% theoretical sulfur content (TSC) [45], which indicated that Br would be a better choice due to its synergetic effect with S on the surface of HSPC for Hg^0^ removal. The mechanism of Hg^0^ adsorption on brominated petroleum cokes was illustrated in Figure 7. It was seen that bromine and sulfur compounds produced a complex. When Hg came in contact with the brominated PC, it formed a Br–Hg–Cn–S–Cn complex. Combined with the XPS analysis, it was these oxidized forms of mercury that were bound on the active sites to form possible species of Cn–Hg, Cn–Hg–Br, Cn–S–Cn–Hg, and/or Cn–S–Cn–Hg–Br. In addition, Marczak-Grzesik et al. [64] pointed out more content of bromine led to the higher Hg^0^ and Hg^2+^ removal performance that Hg^0^ was first oxidized at the activated sites on the adsorbent, followed by Hg^2+^ adsorption. Considering the sulfur in HSPC, especially thiophene, Br would be a promising additive to fully use the inactive organic sulfur on the surface of HSPC for mercury removal.

## 4. Micro-Scale Mechanism of Physical-Chemical Evolution of HSPC Activation and Hg^0^ Removal

### 4.1. DFT Study

Density functional theory (DFT), which is a rigorous theory for solving single electron problems, has been successfully applied for the explanation of mercury binding on carbon-based adsorbents [16]. Due to its calculation principle, which solves the Schrodinger equation with the function of the electron density distribution, the Quantum-level calculations result in a huge computational task that is suitable for a small molecule model rather than a larger one that is always recognized over 100 atoms [65]. Therefore, the selections of appropriate functional, basis sets and calculation methods are crucial to keep the accuracy matching the experimental results and reduce the calculation loading [66]. There exist two assumptions of the adsorption process on the carbon-based surface including plane and spatial adsorption morphology [67]. Plane adsorption occurs when the Hg^0^ atom is put near the unsaturated C, while spatial adsorption occurs there is a lack of or far from the unsaturated C. Furthermore, in the recent two years, some researchers not only proposed the Hg^0^ removal mechanism of activated carbon-based adsorbent but also successfully explored the activation mechanism of raw materials, which promoted the whole process of activation of HSPC and Hg^0^ removal. In addition, the Gaussian [68], a software, is chosen to be the platform for its calculation. 

**Figure 8:**
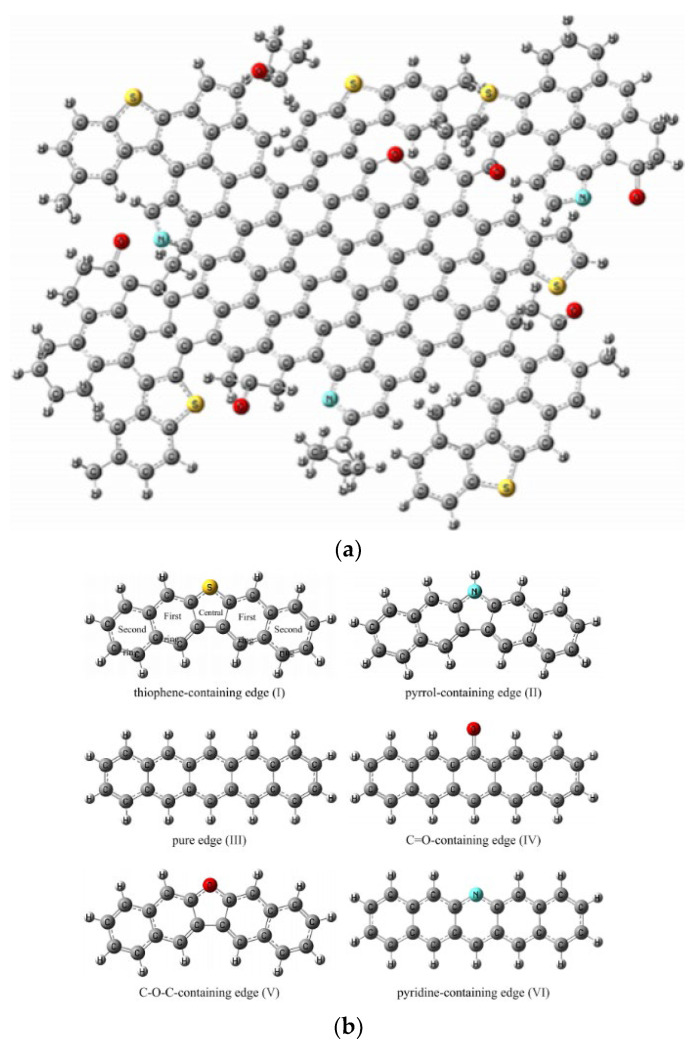
(**a**) Large-scale molecular model of HSPC [69] Reprinted/adapted with permission from Ref. [69]. 2015, Energy and Fuels and (**b**) selected edge model of brominated HSPC [67]. Reprinted/adapted with permission from Ref. [67]. 2019, Energy and Fuels.

Chen Chen et al. [67] built six typical edges of HSPC including thiophene-containing, pyrrole-containing, C=O-containing, C–O–C-containing, pyridine-containing, and pure edges. The brominated process and the complete reaction mechanisms of mercury removal by simulation were presented. As shown in Figure 8, these edge models were selected from the large molecular structure of uncalcined petroleum coke [69]. Generally, the standard to distinguish whether it is physical or chemical adsorption is the adsorption energy of 42 KJ/mol. According to this, the physical adsorption of unburned raw petroleum coke was identified that the Hg^0^ atom was optimized on the above petroleum coke rather than the platform adsorption. In detail, the rank of adsorption energy on petroleum coke was that thiophene-containing edge > pyrrole-containing edge > pure edge > pyridine-containing edge > C–O–C-containing edge > C=O-containing. Although thiophene is so stable to not react with Hg^0^, the thiophene-containing edge would contribute to the physical adsorption of mercury. Furthermore, the Mayer bond of C–S of 1.01 was significantly higher than that of Hg–S of only 0.005, which revealed the stabilization of thiophene on the surface of HSPC. In addition, if some H atoms were removed from C–H to make C-edge active, the calculation result showed the same platform adsorption as that of other carbon-based adsorption and the physical adsorption transformed into chemical adsorption. Especially in the functional group of C=O, the adsorption energy reached up to more than 60 KJ/mol which was higher than 42 KJ/mol. Actually, regardless of revised or fresh petroleum coke, both C–H and active C commonly existed. Thus, a new reaction of spatial reaction pathway and general platform reaction pathway both exist on the surface of petroleum coke for Hg^0^ removal. In terms of Br loading, the adsorption energy of around 355 KJ/mol and Mayer bond of approximately 1 verified that was chemical loading by mechanochemical bromination method which supported the experimental results of Xiao et al. [38]. For the production of HgBr, the C=O edge promoted its generation whose adsorption energy was 291 KJ/mol, which was nearly 20% higher than other edges. Due to the property of inactive C–H and further replacement of C–Br, the spatial reaction pathway of HgBr is general for Br-revised petroleum coke on Hg^0^ removal. In detail, the transition state of mercury oxidation was the process in which the mercury atom rotationally invaded into C–Br and the charge of the mercury atom rose from −0.035 to 0.276 e. The formation of HgBr_2_, where the relative energy was slightly higher than that of HgBr, would exist when there was more Br loading.

**Figure 9:**
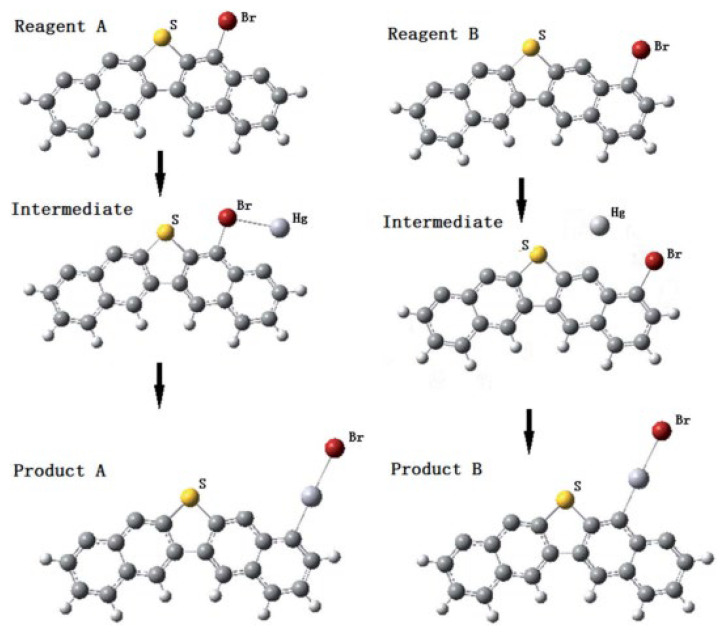
Hg^0^ adsorption process on brominated petroleum coke model with one Br atom at different carbon sites near sulfur [55]. Reprinted/adapted with permission from Ref. [55]. 2021, RSC Advances.

Xiao et al. [55] focused on the effect of thiophene sulfur on petroleum coke for mercury removal. Both experiment and DFT study proved that the mercury adsorption capacity directly correlated with the surface organic sulfur and the binding bromine content. Figure 9 showed the two positions of Br loading. The synergetic effect of the inherent thiophene sulfur and loaded bromine to enhance the mercury removal performance was also found in the DFT study. In detail, the Br atom preferred to bind with the C atom which was beside the thiophene, where the total energy of this setting was 42 KJ/mol higher than other models. Comparing the STE and the C–Hg BMP of the two products, product A was more stable than product B, which indicated that HgBr_2_ could be formed to bind to the carbon site nearest to the S atom. Thus, although thiophene was stable and inactive for Hg^0^ removal in terms of macro performance, the DFT study would help us deeper understand the microscopic chemical reaction mechanism of the mercury removal process on the surface of HSPC.

**Figure 10:**
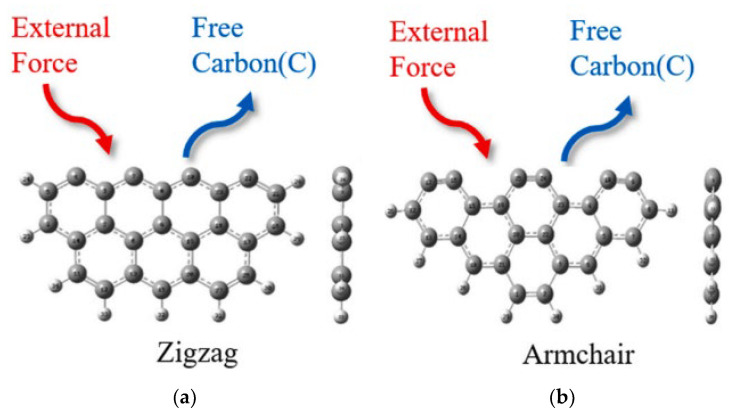
Defective structure caused by external force: (**a**) Zigzag model and (**b**) Armchair model [71]. Reprinted/adapted with permission from Ref. [71]. 2022, Journal of Hazardous Materials.

Apart from HSPC, DFT study has been widely used to explain the mechanisms of preparation of carbon-based adsorbents and their mercury oxidation. The model of graphene and further revised graphene were also commonly selected. Besides this, Stephen A. Hodge et al. [70] proposed the single-walled carbon nanotube molecular structures. As shown in Figure 10, the models of carbon-based adsorbents for mercury removal in some DFT studies in recent years were based on this proposal, including Zigzag (n ≠ 0, m = 0, θ = 0°) and Armchair (n = m ≠ 0, θ = 30°).

Bihter padak et al. [72] selected the graphene model to examine the effect of Cl and the binding performance of Hg, HgCl, and HgCl_2_. The existence of active C led to the adsorption process mainly being the platform reaction rather than the spatial reaction pathway. The Cl atom could strongly bind with C as chemical adsorption like Br [67]. Chompoonut et al. [73] selected a two-row graphene model to reveal the strong effect of halide type on the activation energy barrier of HgX formation, which was in the order of HgI < HgBr < HgCl. Forouzan Vakili et al. [74] created an N, S co-doped graphene model, which was more than three times larger than other studies, to successfully find that Co-doping the graphene led to considerable adsorption energy of −181 KJ/mole on the site close to the sulfur and nitrogen dopants. Jun et al. [75] selected the Zigzag model for mercury removal. The loading of O_2_ and NO under plasma on porous carbon included the functional group of N*, O*, NO*, NO_2_*, and O_3_*. As additives for the raw Zigzag model, all these products by plasma were bound with an active C atom and then oxidize Hg^0^. Particularly, the preadsorbed NO_2_ and O_3_ groups could react directly with Hg to form HgO. 

**Figure 11:**
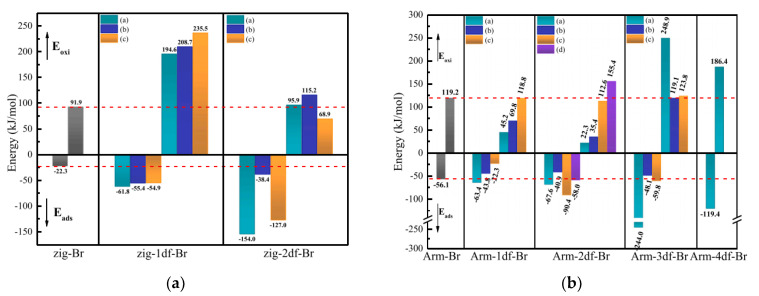
The summary of mercury adsorption energy and oxidation barrier on (**a**) Zigzag, and (**b**) Armchair [71]. Reprinted/adapted with permission from Ref. [71]. 2022, Journal of Hazardous Materials.

Apart from these studies of complete or perfect microstructures as research objects, defective molecular structures also raised some concerns. Zhong et al. [76] design the defective part in the center of the graphene net and showed the chemical adsorption of Al-C while physical adsorption of Hg-C. Furthermore, the adsorption energy of the Hg atom increased with the addition of the Al atom. For comparison, only one Al atom showed the best performance for Hg^0^ adsorption. Compared to the defective structure of opened C atom, Yan et al. [77] investigated both the Zigzag and Armchair models of complete benzene rings lacking one or two carbon atoms. In particular, only semiquinone favored the chemisorption of Hg^0^ because the oxygen atom was also the active site to interact with the Hg^0^ while the other groups showed physisorption of Hg^0^. Geng et al. [71] revealed the mercury removal mechanism of mechanochemical bromination of unburned carbon in fly ash and its mercury removal mechanism based on previous experimental data. Based on the two models (Figure 10), the defective structures lacking carbon atoms were caused by mechanical forces. Compared to the complete models, the energy barrier of the defective one with Br was much lower, especially the Armchair model lacking three carbon atoms, which showed more than 20%. In other words, this calculation result verified the macro-scale performance that the preparation of mechanochemical bromination could improve the Br loading on the unburned carbon. Furthermore, Br-modified defective models could decrease the oxidation energy barrier of Hg^0^ on the surface. The main factor for the high mercury removal efficiency was that the absolute adsorption energy of the appropriate defects is greater than their oxidation energy barrier (Figure 11). It is noted that the parameters, including adsorption energy, energy barrier, mayor bond, etc., of these two models or revised models, showed a significant difference in values and trends. Thus, the selection and construction of the research model is a crucial step in introducing its representative for the calculation of the chemical modification process and mercury adsorption and oxidation.

In addition, the DFT method also can be used to explore the pyrolysis process. Yang et al. [78] explored the decomposition of single thiophene molecular with or without an H atom and the formation of H_2_S during pyrolysis. Three possible pyrolysis pathways were proposed. Among that, the pathway, the furthest H atom transferred to the beside C atom of S atom, showed the lowest energy barrier compared to the homolysis reaction. Also, Li et al. [79] studied the role of SO_2_ and H_2_O in the adsorption process on the ceria surface. This study makes contributions to theoretical research of adsorption environments where exist many poisons including SO_2_, CO, CO_2_, NO_X_, H_2_O, etc.

Overall, the construction of the initial model and the design of the revised model to accurately represent macro-scale samples should be under rigorous argument. The exploration of the reaction pathway could not only distinguish the complex chemical reaction which might not be determined by existing technologies such as SEM, XPS, FTIR, and Hg-TPD but also could propose guidance for the further preparation of mercury removal adsorbent in advance. Thus, DFT is a popular and promising method of calculation for small-scale molecular reactions, including activation of raw petroleum coke, adsorption and oxidation of Hg^0^, and even the effects of composition in flue gas on its anti-toxic performance. 

### 4.2. ReaxFF Study

The reactive force-field (ReaxFF) method for atomistic-scale computational technique trades accuracy for lower computational expense, making it possible to reach simulation scales that are orders of magnitude beyond what is tractable for QM [80]. Based on classical principles, empirical interatomic potentials require significantly fewer computational resources which was the reason why ReaxFF enables to better describe dynamic processes over longer timeframes and on larger scales [81]. ReaxFF is successfully used to conduct the decomposition simulation of large-scale petroleum coke molecular during pyrolysis or combustion process where the greater structural diversity should be detailed considered. The software, which are Materials Studio (MS) and Large-scale Molecular Massively Parallel Simulator (LAMMPS) [82,83], are the general molecular model construction and calculation platforms.

**Figure 12:**
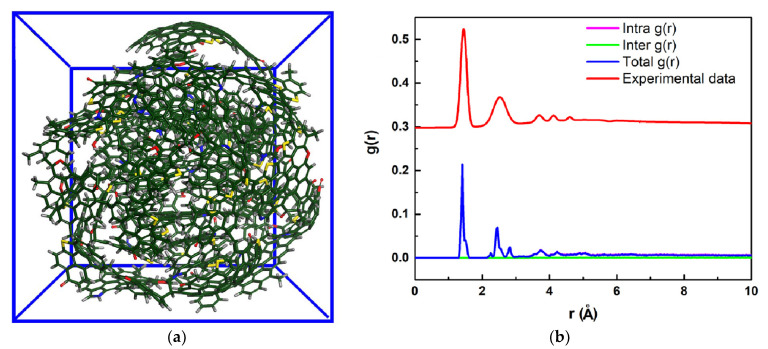
(**a**) Atomic representation (reaction box of 64 × 64 × 64 Å) and (**b**) pair correlation function [84]. Reprinted/adapted with permission from Ref. [84]. 2018, Combustion and Flame.

Xiao et al. [69] constructed and optimized 2D and 3D large-scale molecular models of C_192_H_93_N_3_O_7_S_6_ of raw PC, C_182_H_8_NO_3_S_4_ of a calcined sample under 1300 °C, and C_186_H_8_NO_2_S of a calcined sample under 1400 °C, which were based on and verified by ultimate and XPS analysis. Zhong et al. [84,85] explored the pyrolysis behavior of HSPC for 250 ps at 3000, 3500, and 4000 K with an NVT ensemble, and the model was placed in a reaction box of 64 × 64 × 64 Å, as shown in Figure 12. Thiophene sulfur pyrolyzed from HSPC molecule is presented. In detail, compared with the experimental result of TG-MS, the sulfur removal transformation during pyrolysis was proposed which be thiophene sulfur → COS, C_2_S, or CNS → HS → SO_2_ or CS_2_.

**Figure 13:**
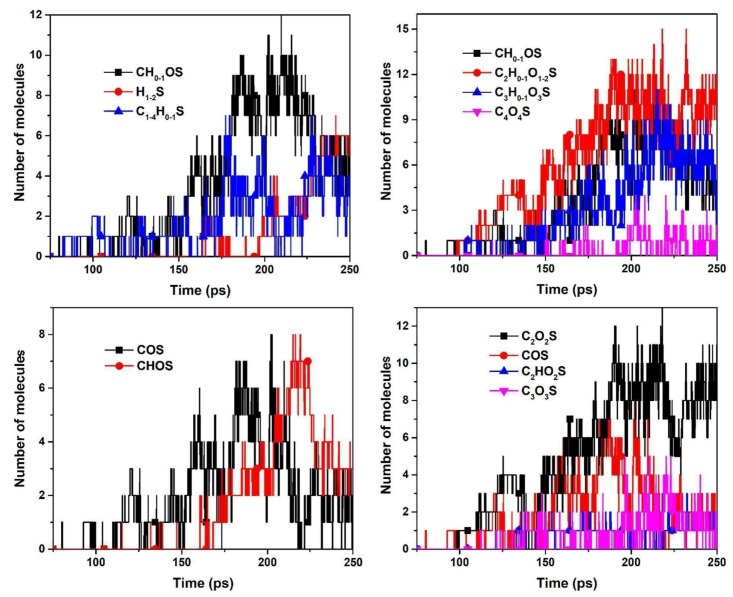
Distributions of the S-containing gaseous products obtained from the CO–petcoke ReaxFF simulations heated to 3000 K [86]. Reprinted/adapted with permission from Ref. [86]. 2019, Fuel.

Zhong et al. [86] further studied the effect of CO, as a reductive gas component, on the S/N removal mechanism of petroleum coke at 3000 K for 250 ps. A significantly larger model, C_1648_H_772_O_59_N_24_S_47_, was constructed under the synchronous pyrolysis and gasification process [85]. In particular, the ReaxFF parameters were obtained and trained based on both experimental and DFT simulation. The Boudouard reaction was observed with one O atom in CO_2_ bonding with a C atom then the O-C bond in CO_2_ breaks after producing CO, and the C atom that bonded with the O atom is then removed to produce the second CO molecule. Thus, it is assumed that CO/CO_2_ reaction with petroleum coke would lead to the loss of C to gas. In particular, as shown in Figure 13, the transformation of S, which we were concerned about being conducive to mercury removal, was as such: thiophenic–S → COS, C_1–2_S → C_n_O_n_S, CO_n_S → H_1–2_S, SO_2_. In discussion, too high pyrolysis temperature could make the inherent S to SO_2_ gas while products of exposure of S from the reserve of sulfur are needed. Meanwhile, an increase in the oxygen-containing functional groups was observed in this study. Thus, it is hopeful to expose and activate the stable S-containing functional groups and increase the O-containing functional groups in the pyrolysis atmosphere which are CO, CO_2_, O_2_, etc. In addition, it is crucial to combine the methods of DFT and ReaxFF to actually present the HSPC for the simulation of activation and further Hg^0^ removal. Furthermore, Zhong et al. [87] introduced the H_2_ and NH_3_ to the desulfurization of HSPC. The result illustrated that under a pyrolysis atmosphere, regardless of H_2_ or NH_3_, the sulfur removal pathway occurred that C_1__–4_–S decomposed and then transformed to H_2_S, which was different from the effects of those reductive atmospheres. Therefore, different pyrolysis atmospheres to pyrolyze HSPC could lead to the different migration forms and pathways of sulfur.

**Figure 14:**
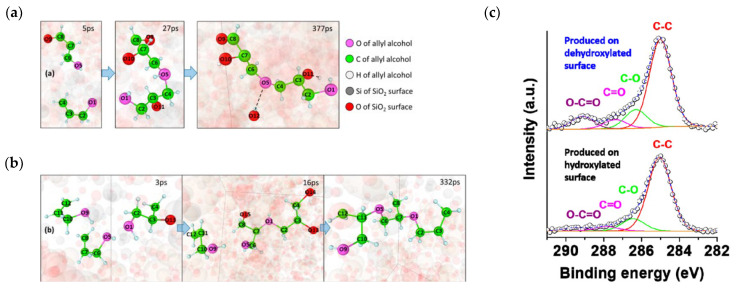
The ReaxFF simulation showing the association paths of (**a**) two and (**b**) three allyl alcohol molecules during sliding at the SiO_2_/SiO_2_ interface (contact pressure = 1 GPa; slide speed = 10 m/s; temperature = 300 K) [88], Reprinted/adapted with permission from Ref. [88]. 2017, Applied Materials. and (**c**) C 1s XPS spectra of the shear-induced polymers produced on silicon oxide surfaces with two different surface conditions [89]. Reprinted/adapted with permission from Ref. [89]. 2018, Langmuir.

Apart from the simulation of pyrolysis, the effects of chemical–mechanical activation on HSPC are also expected to be explained on the micro scale by employing the ReaxFF method. Actually, some ReaxFF studies were carried out on mechanochemistry at solid surfaces caused by shear. Yeon et al. [88] studied the states of polymerization of allyl alcohol adsorbed and sheared at a silicon oxide interface. As shown in Figure 14a,b, the majority of chemical bonds leading to the association of allyl alcohol molecules were formed between the hydroxyl groups of one molecule and one of the carbon atoms in the C=C double bond of another molecule, not between two C=C double bonds as in typical radical polymerization reactions. It suggested that the association reaction pathway of allyl alcohol molecules induced by mechanical shear was quite different from chemically induced polymerization reactions. Moreover, this study mentioned that some degree of distortion of the molecule from its equilibrium state was necessary for mechanically induced chemical reactions. Khajeh et al. [89] explored shear-induced polymerization reactions that occurred during the vapor phase lubrication of α-pinene between sliding hydroxylated and dehydroxylated silica surfaces. It is interesting that these tools of molecular dynamics can present the evolution of functional groups. In this case, the results suggested that oxidative chemisorption of the α-pinene molecules at reactive surface sites, which transferred oxygen atoms from the surface to the adsorbated molecule (Figure 14c), is an critical activation step. In terms of HSPC, mechanochemical activation has been proved to be a promising activation method for Hg^0^ removal. For it, the understanding of the micro-scale mechanism of the activation process is important to find the evaluation of functional groups and also propose some guidance for the experimental and actual application. In addition, limited by the calculation performance of CPU, Li et al. [90] reviewed the advanced MD program using GPU and GMD-Reax [91], which was expected to complete parameter calculations for larger-scale molecular models in less time. In particular, this advanced program has been successfully applied to simulate the pyrolysis and combustion of coal. It means that this method using advanced computational technology would be used for exploration of transformation mechanism of HSPC for Hg^0^ removal.

In summary, ReaxFF is a promising theory to help simulate the decomposition of the larger-scale molecular model of HSPC in the pyrolysis and combustion process. Particularly, the transformation of the S-containing functional group and the generation of the O-containing functional group under different pyrolysis parameters, including atmospheres, such as CO, CO_2_, O_2_, H_2_, etc., final temperature, and heating rate are expected to be studied. Furthermore, ReaxFF studies are also expected to explain the micro-scale chemical–mechanical activation process of HSPC for Hg^0^ removal. Due to the relatively lower calculation accuracy caused by the calculation principle compared to DFT, the combination of DTF and ReaxFF would reduce the calculation time on the premise of ensuring accuracy. Furthermore, a more advanced computational program based on ReaxFF could be introduced to explore the preparation mechanism of HSPC for Hg^0^ removal.

## 5. Conclusions and Further Work

As a low-economic-value by-product of the delayed coking process, HSPC has been a promising choice of carbon-based adsorbent for Hg^0^ removal from coal-fired plants. In terms of the development of pore structure on Hg^0^ adsorption, all activation methods including only and combined chemical, pyrolysis, mechanochemistry, and KOH activation can improve the pore structure of HSPC to a certain extent with micropores. Although the KOH activation can obtain the ideal BET surface of 1713.8 m^2^/g, the undesired loss of inherent sulfur occurred, which is not conducive to the full utilization of HSPC for Hg^0^ oxidation. In addition, it is evident that the big share of the developed mesopores is beneficial for internally decomposed sulfur to load and play an important role to oxidize Hg^0^. It is comprehensive to determine the optimal pore structure due to this coupling effects on both physical adsorption of Hg^0^ and chemicals loading for Hg^0^ oxidation. Among these activation methods, pyrolysis with selected parameters, called controlled pyrolysis, shows its advance and is hoped to improve the pore structure of HSPC, while KOH activation loses almost sulfur content.

The S-containing functional groups on the surface of revised HSPC are crucial to oxidizing Hg^0^ from flue gas. On the one hand, there were few studies paying attention to the exposure of inherent sulfur of HSPC while the inherent sulfur makes the raw materials different and more promising compared to other carbon-based adsorbents represented by commercial activated carbon. It is evident that the distribution of S on the surface and in the bulk of HSPC is significantly different, and the content and share of thiophene, sulfoxide, mercaptan, sulfate, and sulfone should be determined due to their origins. It is noted that the complete decomposition of thiophene on the surface of HSPC should be under over 1200 °C. On the other hand, the S-containing additives increased the S-containing functional groups to oxidize Hg^0^ to generate HgS (both black and red) which were the main product. The element S and other nonoxidized S are the main players in oxidizing Hg^0^. Furthermore, O-containing functional groups can be generated by KOH activation or in the pyrolysis process under the atmosphere of CO_2_/SO_2_, and the created C–O and C–O–O are active for the generation of HgO, while conventional chemical activation could not play well on it. The introduction of Br-additives not only showed its high performance of Hg^0^ removal to generate HgBr and HgBr_2_, but also gave an interesting synergetic effect with S on the surface of HSPC for Hg^0^ removal. 

The technologies of the DFT and ReaxFF methods show their advance in the exploration and explanation of the mechanism of the pyrolysis, chemical activation process, and mercury binding on the surface of modified HSPC. The DFT study concentrated on the edge model, which was less than 100 atoms, of Br loading and Hg^0^ oxidation. Meanwhile, the ReaxFF studies could simulate the pyrolysis with different pyrolysis parameters of a large-scale molecular model of over 1000 atoms based on classical principles. The transformation of thiophene could be concluded as thiophenic–S → COS, C_1__–2_S → C_n_O_n_S, CO_n_S → H_1__–2_S, and SO_2_. Moreover, visualization of the decomposition of the petroleum coke model helps deeply and directly understand the pyrolysis mechanism. 

Thus, some future work on activated HSPC for Hg^0^ removal is proposed below. Firstly, the ideal pore structure of HSPC for mercury adsorption and further chemical activation should be explored and determined. Secondly, the exposure of the inherent sulfur for full utilization of raw materials on mercury oxidation, especially thiophene, should be conducted. To obtain relative functional groups, the combination of controllable pyrolysis and chemical–mechanical activation make up a promising preparation method. Lastly, ReaxFF and DFT studies are expected to explain the micro-scale reaction mechanism of activation with different activation parameters, including both controllable pyrolysis and chemical–mechanical activation, and the Hg^0^ removal. This review is expected to guide further experimental and simulational studies of HSPC on Hg^0^ removal and also actual industry application.

## Figures and Tables

**Table 1:** The total sulfur content of HSPC samples selected from different origins.

Samples	Origins	Sulfur Content wt%	Year and Ref.
HSPC1	Shandong, China	7.06	2019 [37]
HSPC2	Tianjing, China	3.5	2020 [48]
HSPC3	China	6.233	2020 [31]
HSPC4	Jiangxi, China	4.3	2021 [49]
HSPC5	Fushun, Liaoning, China	4.74	2022 [34]
HSPC6	Alberta, Canada	5.85	2017 [38]
HSPC7	Saskatchewan, Canada	2.14	2017 [46]
HSPC8-1	Tianjing, China	7.21	2016 [50]
HSPC8-2	Qingdao, China	6.53	2016 [50]
HSPC8-3	Zhenhai, China	5.21	2016 [50]
HSPC8-4	Dongming, China	2.25	2016 [50]
HSPC9-1	Nanjing, China	2.08	2016 [40]
HSPC9-2	America	6.01	2016 [40]
HSPC10	Jiangsu, China	6.81	2019 [51]
HSPC11	Jamnagar, Gujarat, India	6.31	2019 [52]
HSPC12-1	Zibo, China	3.6	2020 [53]
HSPC12-2	America	3.81	2020 [53]
HSPC13	Canada	6.5	2020 [54]
HSPC14	Xiamen, China	8.92	2021 [2]

**Table 2:** Sulfur forms and their absolute content on the surface of RPC, S600–60-17, FeS600 60-25, and FeS600–60-17 (at. %) [45]. Reprinted/adapted with permission from Ref. [45]. 2022, Fuel Processing Technology.

Samples	Sulfide	Thiophene/ Elemental Sulfur	Sulfoxide	Sulfone	Sulfonate	Sulfate
RPC	3.05	1.56	0.00	0.08	0.00	0.04
S600–60-17	0.61	6.33	3.23	0.72	0.00	0.00
FeS600–60-17	0.52	4.62	2.36	0.00	0.87	0.44
FeS600–60-25	2.82	3.66	1.86	0.66	2.86	1.46

**Table 3:** Surface functional groups obtained by deconvolution of the C 1s XPS Peak [31]. Reprinted/adapted with permission from Ref. [31]. 2020, Energy and Fuels.

Sample	Content of Carbon Functional Groups (Atom %)				Beneficial Oxygen Functional Groups (Atom %)
	C–C/C–H	C–O	C=O	COOH/C(O)–O–C	
RPC	59.54	29.97	0.00	0.00	0.00
CSAC	38.34	11.93	18.93	5.81	24.74
SCAC	16.51	15.24	10.62	19.04	29.66

**Table 4:** The content of elements and carbon-containing functional groups on the surface of the typical BC samples [41]. Reprinted/adapted with permission from Ref. [41]. 2022, Fuel.

Sample	Total Amount (at. %)			Absolute Content (at. %)					
	C	O	N	C–H/C–C	Peak	C=O	Peak	C–O–O–	Peak
BC−N_2_−600−10	87.13	10.61	2.26	75.77	284.80	6.82	286.92	4.55	288.72
BC−N_2_ + 7%O_2_−800−10	88.76	9.55	1.68	73.97	284.80	8.88	286.97	5.92	288.77
BC−N_2_ + 15%CO_2_−700−10	89.21	8.97	1.81	74.97	284.8	8.25	286.88	6.00	288.69

## Data Availability

The data presented in this study are available on request from the corresponding author.

## References

[1] Wang M., Wan Y., Guo Q., Bai Y., Yu G., Liu Y., Zhang H., Zhang S., Wei J. (2021). Brief review on petroleum coke and biomass/coal co-gasification: Syngas production, reactivity characteristics, and synergy behavior. Fuel.

[2] Cai W., Li K., Jiang K., Lv D., Liu Y.-Q., Wang D., Wang X., Lai C. (2021). Utilization of high-sulfur-containing petroleum coke for making sulfur-doped porous carbon composite material and its application in supercapacitors. Diam. Relat. Mater..

[3] Tyutrin A.A., Burdonov A.E., Bushuev K.S. (2022). Expanding the Application Scope of Fine Dust from Petroleum Coke Calcining Furnaces in Aluminum Production. Mater. Sci. Forum.

[4] Veluri P.S., Katchala N., Anandan S., Pramanik M., NarayanSrinivasan K., Ravi B., Rao N.T. (2021). Petroleum Coke as an Efficient Single Carbon Source for High-Energy and High-Power Lithium-Ion Capacitors. Energy Fuels.

[5] Liu L., Li Z., Wu S., Li D., Cai N. (2021). Conversion characteristics of lignite and petroleum coke in chemical looping combustion coupled with an annular carbon stripper. Fuel Process. Technol..

[6] Shan Y., Guan D., Meng J., Liu Z., Schroeder H., Liu J., Mi Z. (2018). Rapid growth of petroleum coke consumption and its related emissions in China. Appl. Energy.

[7] Andrews A., Lattanzio R.K. (2013). Petroleum Coke: Industry and Environmental Issues.

[8] Mackey T.K., Contreras J.T., Liang B.A. (2014). The Minamata Convention on Mercury: Attempting to address the global controversy of dental amalgam use and mercury waste disposal. Sci. Total Environ..

[9] Wang L., Hou D., Cao Y., Ok Y.S., Tack F.M., Rinklebe J., O’Connor D. (2020). Remediation of mercury contaminated soil, water, and air: A review of emerging materials and innovative technologies. Environ. Int..

[10] Streets D.G., Devane M.K., Lu Z., Bond T.C., Sunderland E.M., Jacob D.J. (2011). All-time releases of mercury to the atmosphere from human activities. Environ. Sci. Technol..

[11] Liu K., Wang S., Wu Q., Wang L., Ma Q., Zhang L., Li G., Tian H., Duan L., Hao J. (2018). A highly resolved mercury emission inventory of Chinese coal-fired power plants. Environ. Sci. Technol..

[12] Zhao S., Pudasainee D., Duan Y., Gupta R., Liu M., Lu J. (2019). A review on mercury in coal combustion process: Content and occurrence forms in coal, transformation, sampling methods, emission and control technologies. Prog. Energy Combust. Sci..

[13] Li Y., Yu J., Liu Y., Huang R., Wang Z., Zhao Y. (2022). A Review on Removal of Mercury from Flue Gas Utilizing Existing Air Pollutant Control Devices (APCDs). J. Hazard. Mater..

[14] Li G., Wu Q., Xu L., Wen M., Liu K., Tang Y., Zou J., Wang F., Wang Y., Wang S. (2019). A review on adsorption technologies for mercury emission control. Bull. Environ. Contam. Toxicol..

[15] Beckers F., Rinklebe J. (2017). Cycling of mercury in the environment: Sources, fate, and human health implications: A review. Crit. Rev. Environ. Sci. Technol..

[16] Sasmaz E., Kirchofer A., Jew A.D., Saha A., Abram D., Jaramillo T.F., Wilcox J. (2012). Mercury chemistry on brominated activated carbon. Fuel.

[17] Hu J., Geng X., Duan Y., Zhao W., Zhu M., Ren S. (2020). Effect of mechanical–chemical modification process on mercury removal of bromine modified fly ash. Energy Fuels.

[18] Ochedi F.O., Liu Y., Hussain A. (2020). A review on coal fly ash-based adsorbents for mercury and arsenic removal. J. Clean. Prod..

[19] Zhu C., Duan Y., Wu C.-Y., Zhou Q., She M., Yao T., Zhang J. (2016). Mercury removal and synergistic capture of SO_2_/NO by ammonium halides modified rice husk char. Fuel.

[20] Zafari R. (2021). Synthesis and Study of Modified-Nanocrystalline Cellulose Effective for SO_2_ Capture.

[21] Liu Z., Adewuyi Y.G., Shi S., Chen H., Li Y., Liu D., Liu Y. (2019). Removal of gaseous Hg^0^ using novel seaweed biomass-based activated carbon. Chem. Eng. J..

[22] Yang J., Zhao Y., Zhang J., Zheng C. (2014). Regenerable cobalt oxide loaded magnetosphere catalyst from fly ash for mercury removal in coal combustion flue gas. Env. Sci. Technol..

[23] Liu Z., Liu D., Zhao B., Feng L., Ni M., Jin J. (2020). Mercury Removal Based on Adsorption and Oxidation by Fly Ash: A Review. Energy Fuels.

[24] Wang S., Zhang Y., Gu Y., Wang J., Yu X., Wang T., Sun Z., Romero C.E., Pan W.-p. (2019). Coupling of bromide and on-line mechanical modified fly ash for mercury removal at a 1000 MW coal-fired power plant. Fuel.

[25] Graydon J.W., Zhang X., Kirk D.W., Jia C.Q. (2009). Sorption and stability of mercury on activated carbon for emission control. J. Hazard. Mater..

[26] Yang W., Li Y., Shi S., Chen H., Shan Y., Liu Y. (2019). Mercury removal from flue gas by magnetic iron-copper oxide modified porous char derived from biomass materials. Fuel.

[27] Shen F., Liu J., Dong Y., Wu D. (2018). Mercury removal by biomass-derived porous carbon: Experimental and theoretical insights into the effect of H2S. Chem. Eng. J..

[28] Spessato L., Bedin K.C., Cazetta A.L., Souza I.P., Duarte V.A., Crespo L.H., Silva M.C., Pontes R.M., Almeida V.C. (2019). KOH-super activated carbon from biomass waste: Insights into the paracetamol adsorption mechanism and thermal regeneration cycles. J. Hazard. Mater..

[29] Chen W., Gong M., Li K., Xia M., Chen Z., Xiao H., Fang Y., Chen Y., Yang H., Chen H. (2020). Insight into KOH activation mechanism during biomass pyrolysis: Chemical reactions between O-containing groups and KOH. Appl. Energy.

[30] Wu M.B., Zha Q.F., Qiu J.S., Han X., Guo Y.S., Li Z.F., Yuan A.J., Sun X. (2005). Preparation of porous carbons from petroleum coke by different activation methods. Fuel.

[31] Zhu M., Yan Q., Duan Y., Li J., Zhang X., Han Z., Meng J., Wang S., Chen C., Wei H. (2020). Study on Preparation and Mercury Adsorption Characteristics of Columnar Sulfur-Impregnated Activated Petroleum Coke. Energy Fuels.

[32] She M., Jia C.Q., Duan Y., Zhu C. (2020). Influence of Different Sulfur Forms on Gas-Phase Mercury Removal by SO_2_-Impregnated Porous Carbons. Energy Fuels.

[33] Shen C., Wang H., Shen H., Wu J., Zhu Y., Shi W., Zhang X., Ying Z. (2020). NH4Br-Modified Biomass Char for Mercury Removal in a Simulated Oxy-fuel Atmosphere: Mechanism Analysis by X-ray Photoelectron Spectroscopy. Energy Fuels.

[34] Ma A., Zhao S., Luo H., Sun Z., Xie X., Liao Y., Liang X., Li H. (2022). Mercury removal from coal-fired flue gas of high-sulfur petroleum coke activated by pyrolysis and mechanochemical method. Chem. Eng. J..

[35] Sun Z., Ma A., Zhao S., Luo H., Xie X., Liao Y., Liang X. (2022). Research progress on petroleum coke for mercury removal from coal-fired flue gas. Fuel.

[36] Lee S.H., Rhim Y.J., Cho S.P., Baek J.I. (2006). Carbon-based novel sorbent for removing gas-phase mercury. Fuel.

[37] Huo Q., Wang Y., Chen H., Han L., Wang J., Bao W., Chang L., Xie K. (2019). ZnS/AC sorbent derived from the high sulfur petroleum coke for mercury removal. Fuel Process. Technol..

[38] Xiao Y., Pudasainee D., Gupta R., Xu Z., Diao Y. (2017). Bromination of petroleum coke for elemental mercury capture. J. Hazard. Mater..

[39] Fuente-Cuesta A., Diaz-Somoano M., Lopez-Anton M., Cieplik M., Fierro J., Martínez-Tarazona M. (2012). Biomass gasification chars for mercury capture from a simulated flue gas of coal combustion. J. Environ. Manag..

[40] Li C., Liu X., Zhou Z., Dai Z., Yang J., Wang F. (2016). Effect of heat treatment on structure and gasification reactivity of petroleum coke. Int. J. Coal Sci. Technol..

[41] Zhao S., Luo H., Ma A., Xie W., Sun K., Sun Z. (2022). Influence of pyrolysis conditions on the mercury removal characteristics and physicochemical properties of biomass coke. Fuel.

[42] Hong D., Zhou J., Hu C., Zhou Q., Mao J., Qin Q. (2019). Mercury removal mechanism of AC prepared by one-step activation with ZnCl_2_. Fuel.

[43] Zhao J., Dai Y., Xu J., Chen S., Xie J. (2008). Synthesis and electrochemical characterization of mesoporous carbons prepared by chemical activation. J. Electrochem. Soc..

[44] Adinata D., Daud W.M.A.W., Aroua M.K. (2007). Preparation and characterization of activated carbon from palm shell by chemical activation with K2CO3. Bioresour. Technol..

[45] Ma A., Zhao S., Luo H., Sun Z., Sun K., Li H. (2022). Mercury removal from coal-fired flue gas by the mechanochemical S/FeS modified high sulfur petroleum coke. Fuel Processing Technol..

[46] Xiao Y., Pudasainee D., Gupta R., Xu Z., Diao Y. (2017). Elemental mercury reaction chemistry on brominated petroleum cokes. Carbon.

[47] Zornitta R.L., Barcelos K.M., Nogueira F.G., Ruotolo L.A. (2020). Understanding the mechanism of carbonization and KOH activation of polyaniline leading to enhanced electrosorption performance. Carbon.

[48] She M., Duan Y., Zhu C., Jia C.Q. (2020). Impact of Nonoxidized Sulfur Species on Elemental Mercury Removal by SO_2_ Activated Petroleum Cokes. Energy Fuels.

[49] Yu X., Yu D., Yu G., Liu F., Han J., Wu J., Xu M. (2021). Temperature-resolved evolution and speciation of sulfur during pyrolysis of a high-sulfur petroleum coke. Fuel.

[50] Jin Q.-F.Z.J.X. (2016). Desulfurization of Petroleum Coke by Calcination in Ammonia Atmosphere below 1000 °C. China Pet. Process. Petrochem. Technol..

[51] Wang L., Feng X., Shen L., Jiang S., Gu H. (2019). Carbon and sulfur conversion of petroleum coke in the chemical looping gasification process. Energy.

[52] Tripathi N., Singh R.S., Hills C.D. (2019). Microbial removal of sulphur from petroleum coke (petcoke). Fuel.

[53] Shan J., Huang J.-j., Li J.-z., Li G., Zhao J.-t., Fang Y.-t. (2018). Insight into transformation of sulfur species during KOH activation of high sulfur petroleum coke. Fuel.

[54] Huang Q., Schafranski A.S., Hazlett M.J., Xiao Y., Hill J.M. (2020). Nitric acid functionalization of petroleum coke to access inherent sulfur. Catalysts.

[55] Xiao Y., Liu X., Diao Y. (2021). Understanding the effect of thiophene sulfur on brominated petroleum coke for elemental mercury capture from flue gases. RSC Adv..

[56] D’Alessandro N., Tonucci L., Bonetti M., Di Deo M., Bressan M., Morvillo A. (2003). Oxidation of dibenzothiophene by hydrogen peroxide or monopersulfate and metal–sulfophthalocyanine catalysts: An easy access to biphenylsultone or 2-(2′-hydroxybiphenyl) sulfonate under mild conditions. New J. Chem..

[57] Kumar Reddy K.S., Prabhu A., Al Shoaibi A., Srinivasakannan C. (2016). Application of sulfonated carbons for mercury removal in gas processing. Energy Fuels.

[58] Reddy K.S.K., Al Shoaibi A., Srinivasakannan C. (2018). Mercury removal using metal sulfide porous carbon complex. Process Saf. Environ. Prot..

[59] Abraham A.M., Kumar S.V., Alhassan S.M. (2018). Porous sulphur copolymer for gas-phase mercury removal and thermal insulation. Chem. Eng. J..

[60] Gu Y., Zhang Y., Lin L., Xu H., Orndorff W., Pan W.-P. (2015). Evaluation of elemental mercury adsorption by fly ash modified with ammonium bromide. J. Therm. Anal. Calorim..

[61] Chalkidis A., Jampaiah D., Amin M.H., Hartley P.G., Sabri Y.M., Bhargava S.K. (2019). CeO_2_-Decorated-MnO_2_ Nanotubes: A Highly Efficient and Regenerable Sorbent for Elemental Mercury Removal from Natural Gas. Langmuir.

[62] Dranga B.-A., Lazar L., Koeser H. (2012). Oxidation Catalysts for Elemental Mercury in Flue Gases—A Review. Catalysts.

[63] Hong Y., Duan Y., Zhu C., Zhou Q., She M., Wei H., Hong Y. (2014). Experimental study on mercury removal of high-sulfur petroleum coke activated carbon impregnated with bromine. Proc. CSEE.

[64] Marczak-Grzesik M., Budzyń S., Tora B., Szufa S., Kogut K., Burmistrz P. (2021). Low-Cost Organic Adsorbents for Elemental Mercury Removal from Lignite Flue Gas. Energies.

[65] Obot I., Macdonald D., Gasem Z. (2015). Density functional theory (DFT) as a powerful tool for designing new organic corrosion inhibitors. Part 1: An overview. Corros. Sci..

[66] Makkar P., Ghosh N.N. (2021). A review on the use of DFT for the prediction of the properties of nanomaterials. RSC Adv..

[67] Chen C., Diao Y., Lu Y., Chen S., Tian L. (2019). Complete Reaction Mechanisms of Mercury Binding on Petroleum Coke and Brominated Petroleum Coke. Energy Fuels.

[68] Frisch A. (2009). Gaussian 09W Reference.

[69] Xiao J., Zhong Q., Li F., Huang J., Zhang Y., Wang B. (2015). Modeling the change of green coke to calcined coke using Qingdao high-sulfur petroleum coke. Energy Fuels.

[70] Hodge S.A., Bayazit M.K., Coleman K.S., Shaffer M.S. (2012). Unweaving the rainbow: A review of the relationship between single-walled carbon nanotube molecular structures and their chemical reactivity. Chem. Soc. Rev..

[71] Geng X., Liu X., Ding X., Zhou Q., Huang T., Duan Y. (2022). Mechanochemical bromination of unburned carbon in fly ash and its mercury removal mechanism: DFT study. J. Hazard. Mater..

[72] Padak B., Wilcox J. (2009). Understanding mercury binding on activated carbon. Carbon.

[73] Rungnim C., Promarak V., Hannongbua S., Kungwan N., Namuangruk S. (2016). Complete reaction mechanisms of mercury oxidation on halogenated activated carbon. J. Hazard. Mater..

[74] Vakili F., Rashidi A., Taghavi L., Mansouri N. (2021). Conversion of biomass to N, S co-doped porous graphene as an adsorbent for mercury vapor removal: Optimization and DFT study. J. Environ. Health Sci. Eng..

[75] Liu H., Li J., Xiang K., He S., Shen F. (2021). DFT and Experimental Studies on the Mechanism of Mercury Adsorption on O_2_-/NO-Codoped Porous Carbon. ACS Omega.

[76] Liu Z., Zhang Y., Wang B., Cheng H., Cheng X., Huang Z. (2018). DFT study on Al-doped defective graphene towards adsorption of elemental mercury. Appl. Surf. Sci..

[77] Yan G., Gao Z., Zhao M., Yang W., Ding X. (2020). A comprehensive exploration of mercury adsorption sites on the carbonaceous surface: A DFT study. Fuel.

[78] Yang S., Fan X., Liu J., Zhao W., Hu B., Lu Q. (2021). Mechanism insight into the formation of H_2_S from thiophene pyrolysis: A theoretical study. Front. Environ. Sci. Eng..

[79] Li H., Liu S., Yang J., Liu Y., Hu Y., Feng S., Yang Z., Zhao J., Qu W. (2020). Role of SO_2_ and H_2_O in the mercury adsorption on ceria surface: A DFT study. Fuel.

[80] Chenoweth K., Van Duin A.C., Goddard W.A. (2008). ReaxFF reactive force field for molecular dynamics simulations of hydrocarbon oxidation. J. Phys. Chem. A.

[81] Senftle T.P., Hong S., Islam M.M., Kylasa S.B., Zheng Y., Shin Y.K., Junkermeier C., Engel-Herbert R., Janik M.J., Aktulga H.M. (2016). The ReaxFF reactive force-field: Development, applications and future directions. Npj Comput. Mater..

[82] Sharma S. (2019). Molecular Dynamics Simulation of Nanocomposites Using BIOVIA Materials Studio, Lammps and Gromacs.

[83] Sharma S., Kumar P., Chandra R. (2019). Applications of BIOVIA materials studio, LAMMPS, and GROMACS in various fields of science and engineering. Molecular Dynamics Simulation of Nanocomposites Using BIOVIA Materials Studio, Lammps and Gromacs.

[84] Zhong Q., Mao Q., Xiao J., van Duin A.C., Mathews J.P. (2018). ReaxFF simulations of petroleum coke sulfur removal mechanisms during pyrolysis and combustion. Combust. Flame.

[85] Zhong Q., Mao Q., Xiao J., van Duin A., Mathews J.P. (2018). Sulfur removal from petroleum coke during high-temperature pyrolysis. Analysis from TG-MS data and ReaxFF simulations. J. Anal. Appl. Pyrolysis.

[86] Zhong Q., Zhang Y., Shabnam S., Mao Q., Xiao J., van Duin A.C., Mathews J.P. (2019). ReaxFF MD simulations of petroleum coke CO2 gasification examining the S/N removal mechanisms and CO/CO_2_ reactivity. Fuel.

[87] Zhong Q., Zhang Y., Shabnam S., Xiao J., Van Duin A.C., Mathews J.P. (2019). Reductive gaseous (H_2_/NH_3_) desulfurization and gasification of high-sulfur petroleum coke via reactive force field molecular dynamics simulations. Energy Fuels.

[88] Yeon J., He X., Martini A., Kim S.H. (2017). Mechanochemistry at solid surfaces: Polymerization of adsorbed molecules by mechanical shear at tribological interfaces. ACS Appl. Mater. Interfaces.

[89] Khajeh A., He X., Yeon J., Kim S.H., Martini A. (2018). Mechanochemical association reaction of interfacial molecules driven by shear. Langmuir.

[90] Li X., Zheng M., Ren C., Guo L. (2021). ReaxFF molecular dynamics simulations of thermal reactivity of various fuels in pyrolysis and combustion. Energy Fuels.

[91] Mo Z., Li X., Li G. (2013). Algorithms of GPU-enabled reactive force field (ReaxFF) molecular dynamics. J. Mol. Graph. Model..

